# Venturing Past Uranium: Synthesis of a Np(IV) Polyoxomolybdate–Alkoxide Sandwich Complex

**DOI:** 10.1021/acs.inorgchem.4c04428

**Published:** 2024-11-20

**Authors:** Leyla R. Valerio, Dominic Shiels, Lauren M. Lopez, Andrew W. Mitchell, Matthias Zeller, Suzanne C. Bart, Ellen M. Matson

**Affiliations:** † Department of Chemistry, University of Rochester, Rochester, New York 14627, United States; ‡ H. C. Brown Laboratory, James Tarpo Jr. and Margaret Tarpo, Department of Chemistry, Purdue University, West Lafayette, Indiana 47907, United States

## Abstract

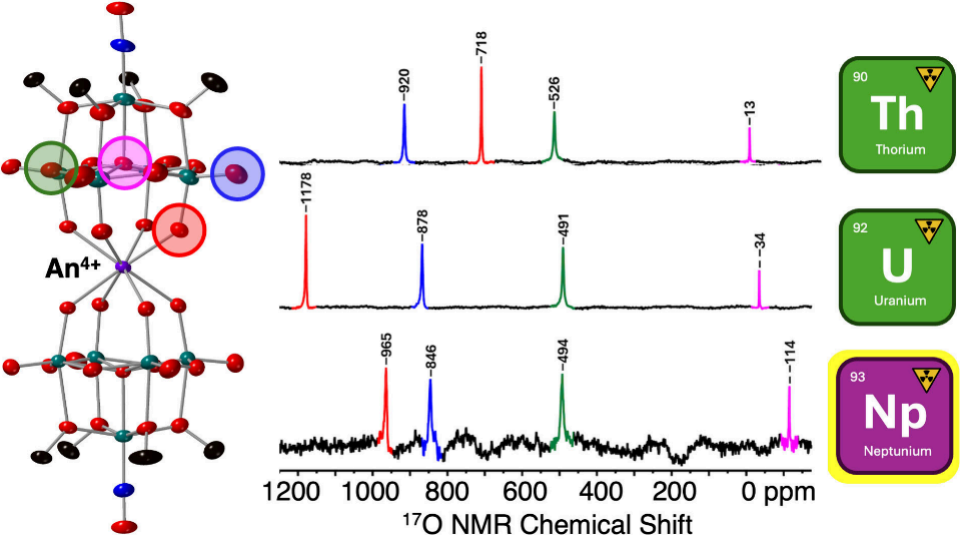

The synthesis of a Np(IV) polyoxomolybdate–alkoxide sandwich complex, (TBA)_2_[Np{Mo_5_O_13_(OMe)_4_NO}_2_] (TBA = tetrabutylammonium), is reported. This compound represents a rare example of a neptunium polyoxometalate cluster isolated outside of water, allowing for characterization of its electrochemical properties in nonaqueous solvents. Complexation of An(IV) cations fine-tunes the redox properties of the cluster, with the observed four reversible reductive events varying slightly both in potential and peak separation depending on the actinide present. The new Np(IV) complex also shows an irreversible event assigned to oxidation of Np(IV) to Np(V). New methodology for facile ^17^O enrichment of (TBA)_2_[Mo_5_O_13_(OMe)_4_NO][Na(MeOH)] is presented, which provides a simple pathway to ^17^O enriched analogues of the sandwich complexes discussed (Zr(IV), Hf(IV), Th(IV), U(IV), U(V), Np(IV)). ^17^O NMR spectroscopy subsequently provides insights into both the nature of metal–oxygen bonding, as well as the influence of unpaired f-electrons on the local environment of the oxygen nuclei.

## Abstract 2

Reaction of a lacunary polyoxomolybdate−alkoxide with neptunium(IV) results in the formation of a rare neptunium−polyoxometalate complex isolated outside of water.

## Introduction

There has been a renewed interest in studying the fundamental chemistry of actinides due to their importance in fields such as spent nuclear fuel processing and environmental remediation.^1−4^ The majority of studies have focused on uranium and thorium, mainly due to their prevalence as carbon neutral fuel sources and the ease of handling their weakly radioactive ^238^U and ^232^Th isotopes. In comparison, less progress has been achieved for transuranium elements, which are present in minor amounts in spent nuclear fuels. This class of elements has reduced availability compared to U/Th, and requires radiological facilities to safely handle these higher specific-activity α-emitters. The chemistry of neptunium (^237^Np),^5^ however, offers an opportunity to study transuranium elements with relatively few obstacles compared to its later actinide counterparts. This element has gained popularity in recent years because of its exciting solution-phase redox chemistry that can span oxidation states of +2 to +7, stabilized using a variety of ligand frameworks, similar to the earlier actinides.

Recently, polyoxometalates (POMs), which are anionic molecular metal oxide clusters typically made from tungsten(VI), molybdenum(VI), or vanadium(V), have emerged as suitable frameworks for the formation of molecular actinide complexes.^6−13^ The stability and ease by which these clusters crystallize has facilitated detailed structural characterization, and for comparisons to be made with transition metal- or lanthanide-containing derivatives.^14−17^ Moreover, the high molecular weight (∼1,000 to 20 000 g/mol) of POMs allows for stoichiometric reactions to be carried out with exceptionally small quantities of actinide starting materials, a property which is especially important when looking to study the highly radioactive and rare transuranium elements.^18,19^

Structurally characterized transuranium POM complexes that leverage polyoxotungstates clusters as “ligands” have been established. Early efforts in this space utilized trilacunary polyoxometalate anions to complex high-valent neptunyl and plutonyl cations.^20,21^ For example, 2:2 or 2:3 cluster complexes incorporating [UO_2_]^2+^, [NpO_2_]^2+^, [NpO_2_]^+^, and [PuO_2_]^2+^ into a polyoxotungstate framework have been reported, forming complexes of the type [Na_2_(AnO_2_)_2_(α-EW_9_O_34_)_2_]^*n*–^ (An = U, *n* = 12; An = Np, *n* = 14) and K_11_[K_3_(PuO_2_)_3_(GeW_9_O_34_)_2_]·12H_2_O.^22−24^ More recently, lacunary POM complexes incorporating transuranium cations with lower charges have been accessed, though many have only been identified in solution-phase studies due to the radioactivity of the heavier transuranium elements.^25−27^ Moisy and co-workers detail structural characterization of a series of An(IV) sandwich complexes using the heteropolyanion [P_2_W_17_O_61_]^10−^ to form An(P_2_W_17_O_61_)_2_^16−^ (An = Th, U, Np, and Pu).^18^ Structural and spectroscopic characterization has also been obtained for a mixed-ligand polyoxometalate complex of Np(IV), which leverages crystallization from a mixture of [Np(W_5_O_18_)_2_]^8–^ and [Np(BW_11_O_39_)_2_]^14–^ to form K_10.5_H_0.5_[Np(BW_11_O_39_)(W_5_O_18_)]·15H_2_O.^6^ Seminal work from Deblonde and co-workers utilizes heteropolyoxotungstate clusters to complex microgram quantities of Am(III) and Cm(III) cations, with structural and spectroscopic characterization obtained for the complexes.^19^

Despite the relative prevalence of literature detailing polyoxotungstate actinide complexes, far fewer reports utilize molybdenum analogues, attributed to the lower stabilities of lacunary polyoxomolybdates.^28^ Interestingly, however, a subset of actinide literature details the role of molybdenum in spent nuclear fuel reprocessing and how the chemical behavior of neptunyl(V) is affected by the molybdate ion.^29,30^ Given understanding the interactions between molybdate ions and neptunium is relevant to energy and the environment, we proposed utilizing a polyoxomolybdate cluster as a ligand for neptunium would provide fundamental insights into the bonding, redox reactivity, and electronic communication between the cluster scaffold and Np ion.

**Figure 1:**
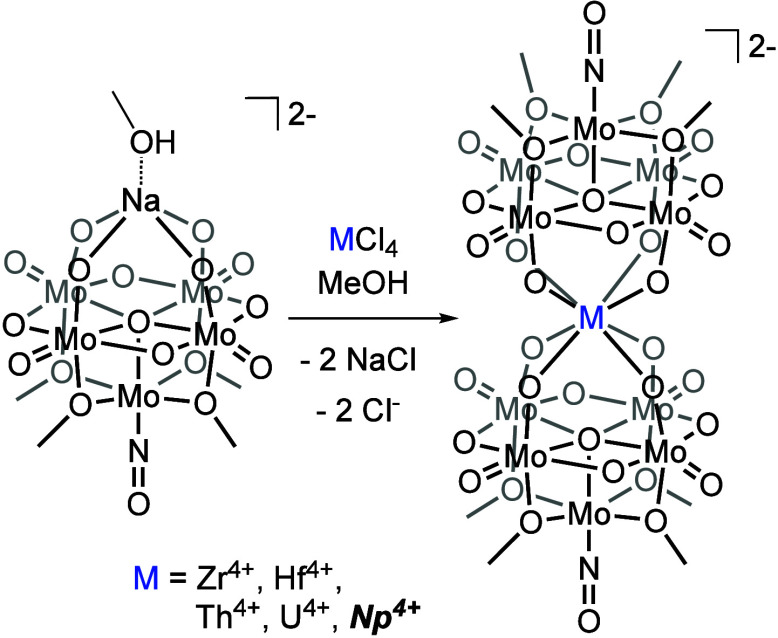
General schematic for the synthesis of sandwich complexes from **1-NaMo**_**5**_, extended to Np(IV) in this work.^31^

Our research team has previously described the synthesis and characterization of a series of polyoxomolybdate–alkoxide sandwich complexes of the general formula, (TBA)_2_[M{Mo_5_O_13_(OMe)_4_NO}_2_] (TBA = tetrabutylammonium), incorporating M(IV) cations (M = Zr(IV), Hf(IV), Th(IV), U(IV)), herein referred to as **2-Zr(Mo**_**5**_**)**_**2**_, **3-Hf(Mo**_**5**_**)**_**2**_, **4-Th(Mo**_**5**_**)**_**2**_, and **5-U(Mo**_**5**_**)**_**2**_.^31^ Complexation of An(IV) ions enhanced the redox properties of the cluster, with the complexes reversibly accepting up to four electrons. It was hypothesized that the large size of the actinide cations was important, as increasing the separation between the {Mo_5_} subunits appears to stabilize the reduced complexes by minimizing intramolecular repulsion. We were intrigued to continue with these studies by examining the effect that a smaller Np(IV) ion would have on the overall electronic structure and redox properties of this family. Herein, we extend the series of M(IV) containing sandwich complexes, generating [Np{Mo_5_O_13_(OMe)_4_NO}_2_]^2–^**7-Np(Mo**_**5**_**)**_**2**_ (Figure 1). Characterization by single crystal X-ray diffraction (SCXRD) reveals that the bond metrics of the neptunium derivative lie between the transition metal and larger actinide derivatives. ^17^O enrichment and analysis by ^17^O NMR spectroscopy of the M(IV) series provides insight into the nature of the M–O bonding and the influence of unpaired f-electrons on the oxygen nuclear environments. Examination of the electrochemical properties of **7-Np(Mo**_**5**_**)**_**2**_ reveal subtle differences in the polyoxoalkoxide-based redox processes when compared to the other sandwich complexes, exemplifying the ability of actinides to influence ligand based redox events. Cyclic voltammetry reveals an irreversible oxidation of Np(IV) to Np(V) in (TBA)_2_[Np{Mo_5_O_13_(OMe)_4_NO}_2_], occurring at 1.54 V, with the presence of an irreversible cluster based oxidation occurring at a similar potential. These are proposed to contribute to the instability of the Np(V) derivative compared to the previously reported U(V) compound.^31^

## Experimental Section

### General Considerations

All air- and moisture-sensitive manipulations with neptunium were performed using standard Schlenk techniques or in an MBraun negative pressure UHP argon atmosphere drybox. All air- and moisture-sensitive manipulations with thorium or uranium were carried out using a standard high-vacuum line, Schlenk techniques, or an MBraun inert atmosphere drybox containing an atmosphere of purified dinitrogen. The MBraun glovebox was equipped with a cold well designed for freezing samples in liquid nitrogen as well as a −35 °C freezer for cooling samples and crystallizations. Solvents for sensitive manipulations were dried and deoxygenated using literature procedures with a Seca solvent purification system or a glass contour solvent purification system (Pure Process Technology, LLC) and stored over activated 4 Å molecular sieves (Fisher Scientific) prior to use. Deuterated solvents were purchased from Cambridge Isotope Laboratories, dried with molecular sieves, and degassed by three freeze–pump–thaw cycles. 40% ^17^O enriched H_2_O was purchased from CortecNet and used as received. (TBA)_4_[Mo_8_O_26_],^32^ (TBA)_2_[Mo_5_O_13_(OMe)_4_NO][Na(MeOH)] (**1-NaMo**_**5**_),^33^ and NpCl_4_(DME)_2_^34^ were synthesized according to literature procedures.

### Safety Considerations

***Caution!***^*237*^*Np represents a health risk due to its α and γ emission and its decay to the short-lived*^*233*^*Pa isotope (t*_*1/2*_*= 27.0 days), which is a strong β and γ emitter. All studies with Np were conducted in a laboratory equipped for radioactive materials. All studies were modeled on depleted uranium prior to working with*^*237*^*Np. Depleted uranium (primary isotope*^*238*^*U) is a weak α-emitter (4.197 MeV) with a half-life of 4.47 × 10*^*9*^*years, and*^*232*^*Th is a weak α-emitter (4.082 MeV) with a half-life of 1.41 × 10*^*10*^*years; manipulations and reactions should be carried out in monitored fume hoods or in an inert atmosphere drybox in a radiation laboratory equipped with α and β counting equipment.*

*The scarcity of neptunium in combination with the relatively high specific radioactivity of*^*237*^*Np requires syntheses to be performed on small scales (<15 mg Np). Fortunately, the high molecular weight of polyoxometalates allows for stoichiometric reactions to be performed with exceptionally small quantities of actinide starting materials, making extension of this chemistry to neptunium favorable. To ensure this, reactions using optimized quantities of depleted uranium as a model for neptunium were undertaken and proved to be successful.*

### Synthesis of (TBA)_2_[Np{Mo_5_O_13_(OMe)_4_NO}_2_] (7-Np(Mo_5_)_2_)

NpCl_4_(DME)_2_ (10 mg, 0.018 mmol, 1 equiv; DME = dimethoxyethane) was weighed into a 5 mL vial and sealed with a septum cap before being taken out of the glovebox. In a separate 20 mL vial, (TBA)_2_[Mo_5_O_13_(OMe)_4_NO][Na(MeOH)] (49 mg, 0.035 mmol, 2 equiv) was dissolved in methanol (MeOH, 3 mL) in air and added dropwise to the solid NpCl_4_(DME)_2_ with a syringe. A green-yellow precipitate formed immediately upon addition. The suspension was gently shaken for 5 min before being opened to air and filtered over Celite in a glass pipette plugged with a microfiber glass filter. A fine green powder was collected on the Celite bed and washed with MeOH (5 mL). The product was extracted with dichloromethane (DCM) until washings ran clear (5 mL). The solvent was removed under a N_2_ flow and the product was subsequently dried under reduced pressure overnight before being brought into the glovebox. Yield: 38 mg, 0.016 mmol, 90%. Yellow-green single crystals were obtained by vapor diffusion of Et_2_O into a saturated solution of the product in acetonitrile (MeCN) at room temperature. ^1^H NMR (400 MHz, CDCl_3_): δ 4.73 (s, 24H), 3.10 (s, 16H), 1.61 (s, 16H), 1.29 (s, 16H), 1.02 (m, 24H). ^17^O NMR (54.2 MHz, CDCl_3_): δ 114.3 (μ_5_-O), 493.9 (Mo–O–Mo), 846.4 (Mo=O), 964.9 (Mo–O–Np). λ_max_ (DCM) = 763 nm (ε = 158 M^–1^ cm^–1^), 900 nm (ε = 92 M^–1^ cm^–1^), 990 nm (ε = 56 M^–1^ cm^–1^), 1,240 nm (ε = 24 M^–1^ cm^–1^), and 1,390 nm (ε = 25 M^–1^ cm^–1^). Broad and intense absorption also observed from 400 to 700 nm.

### ^17^O Enrichment of (TBA)_2_[Mo_5_O_13_(OMe)_4_NO][Na(MeOH)(H_2_O)]

(TBA)_2_[Mo_5_O_13_(OMe)_4_NO][Na(MeOH)] (100 mg, 0.07 mmol, 1 equiv) was dissolved in anhydrous MeOH (5 mL) forming a purple solution. 40% ^17^O enriched H_2_O (6.5 μL, 0.36 mmol, 5 equiv) was added and the solution was stirred at 50 °C for 3 h. The solution was allowed to cool temperature and then the volatiles were removed under vacuum. ^17^O NMR spectroscopy of the crude material showed successful ^17^O enrichment but also revealed the presence of a significant amount of residual water. Therefore, the material was purified by recrystallization. Vapor diffusion of Et_2_O into a saturated solution of the crude material dissolved in anhydrous MeOH led to the formation of large purple crystals. The mother liquor was decanted, washed with Et_2_O (can lead to loss of crystallinity), and dried under vacuum overnight (73 mg, 72% yield). ^1^H NMR (500 MHz, CD_3_OD): δ 1.03 (t, *J* = 7.3 Hz, 24 H), 1.44 (h, *J* = 7.4 Hz, 16 H), 1.68 (m, 16 H), 3.26 (m, 16 H), 4.65 (−OMe), 4.87 (H_2_O)*.*^17^O NMR (67.8 MHz, CD_3_OD): δ −38.8 (Na–OH_*2*_), −15.6 (H_*2*_O), 18.3 (μ_5_-O), 417.2 (Mo–O–Mo), 800.7 (Mo–O–Na), 837.4 (Mo=O). Anal. Calcd for C_37_H_90_N_3_O_19_Mo_5_Na (mol wt 1399.868 g mol^–1^): C, 31.75%; H 6.48%; N, 3.00%. Found: C, 32.03%; H 6.25%; N, 3.37%.

It was noted that the material also readily loses ^17^O enrichment upon dissolution in wet MeOH, presumably due to O atom exchange with the H_2_O present in the solvent. Therefore, to avoid loss of enrichment, the reaction, purification, and subsequent reactions should all be performed in anhydrous solvents where possible.

^17^O enriched analogues of (TBA)_2_[M{Mo_5_O_13_(OMe)_4_NO}_2_] (M = Zr(IV), Hf(IV), Th(IV), U(IV), Np(IV)) were prepared according to methods described previously (and below), using the ^17^O enriched material prepared here.^31^ Characterization data were consistent with what was previously described,^31^ while ^17^O NMR spectra are shown in Figure 6 and Figures S10–S15. A modified method was used to prepare the U(V) containing analogue and is described below.

### Direct Synthesis of (TBA)[U(V){Mo_5_O_13_(OMe)_4_NO}_2_] (6-U(Mo_5_)_2_)

In a 20 mL scintillation vial, (TBA)_2_[Mo_5_O_13_(OMe)_4_NO][Na(MeOH)] (100 mg, 0.07 mmol, 2 equiv) was dissolved in MeCN (5 mL). The purple solution was added to solid UCl_4_ (14 mg, 0.036 mmol, 1 equiv) in a separate vial with stirring. This led to an immediate formation of a dark brown solution. The mixture was stirred for 10 min before adding to a separate vial containing solid [NO][PF_6_] (63 mg, 0.36 mmol, 10 equiv). A brown suspension formed immediately which was stirred for 3 min before passing through a bed of Celite. The solid was washed with a small amount of MeCN (2 × 1 mL) and then extracted with DCM until the washing ran clear (approximately 10 mL). The volatiles were removed under vacuum to leave a dark brown solid (41 mg, 57% yield). ^17^O NMR (67.8 MHz, CD_2_Cl_2_) δ 76.2 (μ_5_-O), 569.0 (Mo–O–Mo), 940.4 (Mo–O–U), 960.0 (Mo=O). Additional characterization was in line with what was previously reported.^31^

#### Physical Measurements

^1^H NMR (NMR = nuclear magnetic resonance) spectra for neptunium compounds were recorded at room temperature on a Bruker AV-III-HD-400 spectrometer operating at 400.13 MHz. All chemical shifts are reported relative to ^1^H residual chemical shifts of chloroform-*d* (7.24 ppm). ^1^H NMR spectra for all other compounds (Th, U) were recorded at room temperature on a 400 MHz Bruker AVANCE spectrometer or a 500 MHz Bruker AVANCE spectrometer locked on the signal of deuterated solvents. All chemical shifts are reported relative to the chosen deuterated solvent as a standard. ^17^O NMR were collected at room temperature on a Bruker AV-III-HD-400 spectrometer (at 54.2 MHz) or a 500 MHz Bruker AVANCE spectrometer (at 67.8 MHz), with the spectrometer locked on the signal of the deuterated solvents and all chemical shifts given relative to an external standard of D_2_O. Cyclic voltammetry (CV) for the neptunium complex was performed using a three-electrode setup inside a negative-pressure argon glovebox (MBraun UniLab, USA) using a CH Instrument 620E potentiostat. The concentration of the cluster and the supporting electrolyte (TBAPF_6_) were kept at 1 and 100 mM, respectively, throughout all measurements. CVs were recorded using a 3 mm diameter glassy carbon working electrode (CH Instruments, USA), a Pt wire auxiliary electrode (CH Instruments, USA), and a silver wire quasi-reference electrode. Ferrocene was used as an internal standard after completion of the measurements, and potentials were referenced versus the Fc^+/0^ couple. Electronic absorption measurements were recorded inside a negative pressure drybox at room temperature in anhydrous MeCN in sealed 1 cm quartz cuvettes using a JASCO V-770 UV–vis–NIR spectrophotometer equipped with a fiber optic stage and sample holder for **7-Np(Mo**_**5**_**)**_**2**_.

#### X-ray Crystallography

Single crystals suitable for X-ray diffraction were coated with poly(isobutylene) oil in the glovebox and quickly transferred to the goniometer head of a Bruker Quest diffractometer with a fixed chi angle, a sealed tube fine focus X-ray tube, single crystal curved graphite incident beam monochromator, a Photon II area detector and an Oxford Cryosystems low temperature device. Examination and data collection were performed with Mo Kα radiation (λ = 0.71073 Å) at 150 K.

## Results and Discussion

**Figure 2:**
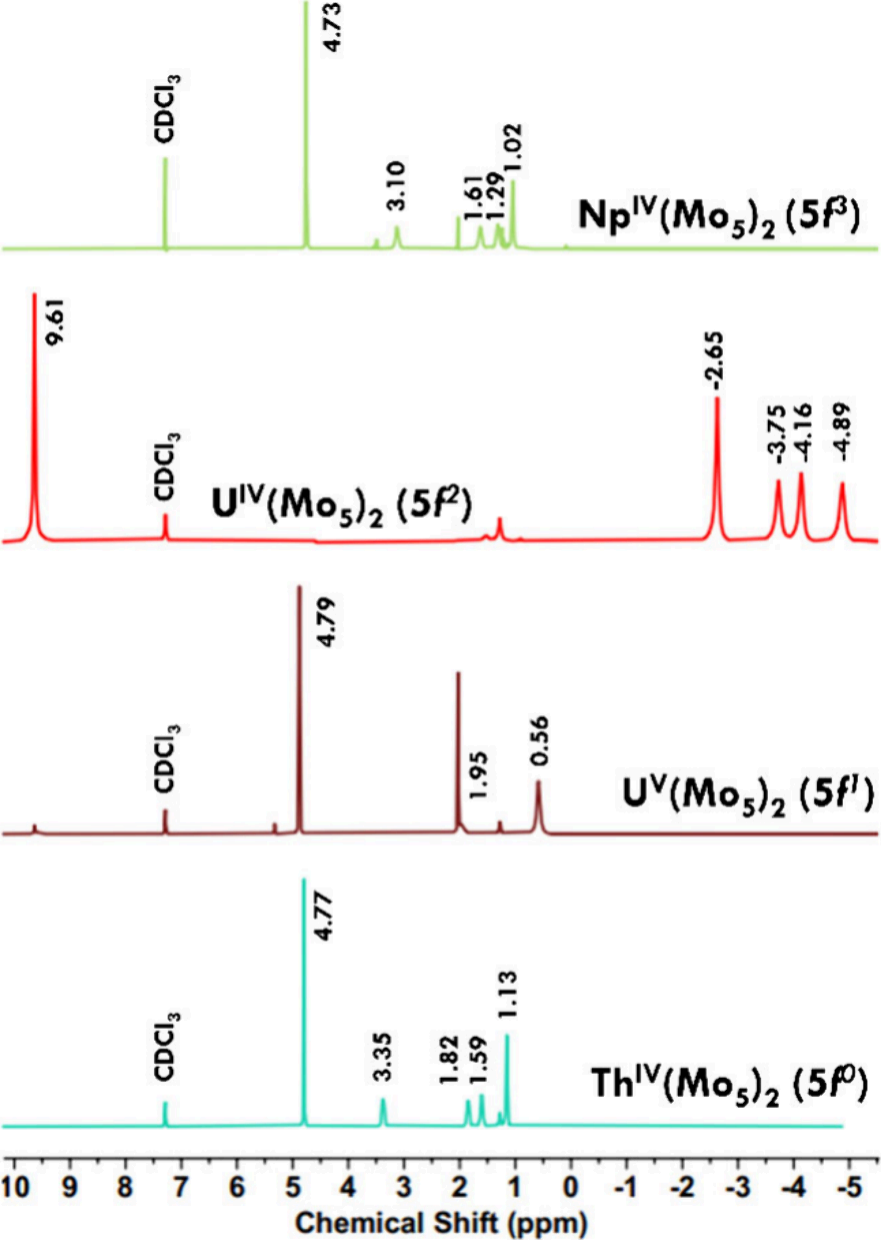
^1^H NMR spectra of **4-Th(Mo**_**5**_**)**_**2**_, **5-U(Mo**_**5**_**)**_**2**_, **6-U(Mo**_**5**_**)**_**2**_, and **7-Np(Mo**_**5**_**)**_**2**_ ordered by increasing 5f electron count. Spectra recorded in CDCl_3_ at room temperature.

To expand upon prior work with actinide(IV) cations, a Np(IV) adduct of the lacunary polyoxomolybdate–alkoxide cluster (TBA)_2_[Mo_5_O_13_(OMe)_4_NO][Na(MeOH)] (**1-NaMo**_**5**_) was synthesized. The addition of a purple solution of **1-NaMo**_**5**_ in MeOH to half an equivalent of pink NpCl_4_(DME)_2_ results in the immediate precipitation of a fine green solid, which is isolated by filtration and extraction with DCM to afford (TBA)_2_[Np{Mo_5_O_13_(OMe)_4_NO}_2_] (**7-Np(Mo**_**5**_**)**_**2**_) (see Experimental Section for additional details). Analysis of the solid via ^1^H NMR spectroscopy reveals a set of four resonances from +3 to +1 ppm, assigned to the associated TBA cations. An additional resonance at 4.73 ppm is assigned to the bridging alkoxide functionalities of the [Mo_5_O_13_(OMe)_4_NO]^3–^ fragment (Figure 2).

To our surprise, the ^1^H NMR spectrum obtained from the reaction with Np(IV) is nearly identical to that acquired for the Th(IV) (5f^0^) analogue despite the former having three unpaired electrons. The spectrum is also drastically different from the U(IV) (5f^2^) sandwich complex, where the paramagnetism of the actinide center results in TBA resonances that appear from −2.5 to −5 ppm as well as a resonance assigned to the methoxide groups of the cluster at 9.61 ppm. In our original report, ^1^H DOSY NMR spectroscopy revealed that for **5-U(Mo**_**5**_**)**_**2**_ the anionic cluster unit and TBA cations were tight ion pairs in solutions with high dielectric constants (strongest in CDCl_3_), leading to the large magnetic interactions that were observed.^31^ It was initially postulated that a neptunium derivative would accordingly display similar or more intense magnetic interactions compared to **5-U(Mo**_**5**_**)**_**2**_, as a consequence of the 5f^3^ electron configuration of the neptunium ion. Rather, the ^1^H NMR spectrum of **7-Np(Mo**_**5**_**)**_**2**_ resembles its diamagnetic congeners, which serves as an initial indication that the protons within the complex are magnetically decoupled from the paramagnetic actinide center. It is important to note that the ^1^H NMR spectrum of **7-Np(Mo**_**5**_**)**_**2**_ is also similar to that of the U(V) (5f^1^) analogue, suggesting that there are likely multiple factors that dictate the extent of observed paramagnetic shifting within the complexes (e.g., electronic configuration, cation size, distance of the protons from the center). Evidently, the actinide center within these systems has a significant impact on the local environment of the protons within these complexes, and **5-U(Mo**_**5**_**)**_**2**_ likely has unique magnetic properties that are significantly different from the other isolated derivatives.

**Figure 3:**
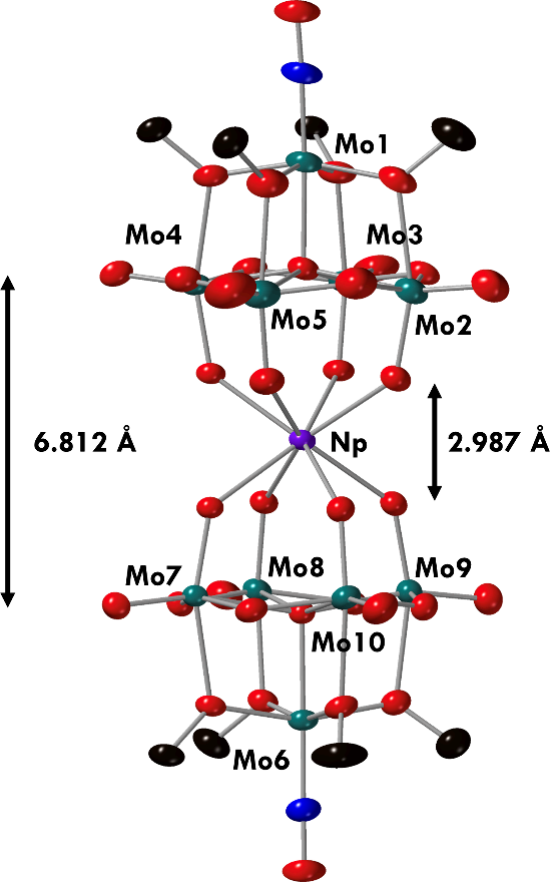
Single crystal X-ray diffraction structure of **7-Np(Mo**_**5**_**)**_**2**_ with probability ellipsoids set at 50%. The tetrabutylammonium cations and some disorder has been masked for clarity.

To further characterize the product, crystals of **7-Np(Mo**_**5**_**)**_**2**_ suitable for single crystal X-ray diffraction (SCXRD) were grown by vapor diffusion of diethyl ether into a saturated solution of the product in acetonitrile at room temperature. Refinement of the data confirmed the structural composition of **7-Np(Mo**_**5**_**)**_**2**_ as the eight-coordinate species (TBA)_2_[Np{Mo_5_O_13_(OMe)_4_NO}_2_] (Figure 3, Table 1). The solid-state structure features a central neptunium ion in a square antiprismatic geometry, sandwiched between two cluster units. The average bond distances between the molybdenum and oxygen atoms within the [Mo_5_O_13_(OMe)_4_NO]^3–^ unit are consistent with those observed in the transition metal(IV), Th(IV), and U(IV) congeners, highlighting that incorporation of either transition metal or actinide ions does not cause significant perturbations in the solid-state molecular structure of the cluster units. The average M–O bond lengths of approximately 2.35 Å for **7-Np(Mo**_**5**_**)**_**2**_ are shorter than those of **4-Th(Mo**_**5**_**)**_**2**_ and **5-U(Mo**_**5**_**)**_**2**_ (2.41 and 2.36 Å, respectively), but longer than **2-Zr(Mo**_**5**_**)**_**2**_ and **3-Hf(Mo**_**5**_**)**_**2**_ (2.20 and 2.19 Å, respectively). This can be rationalized on the basis of the effective ionic radius of the Np(IV) cation (0.98 Å),^35^ which is larger than the transition metal derivatives, but smaller than the previously isolated actinide clusters (Hf < Zr ≪ Np < U < Th). This is likely the result of increasing nuclear charge moving from left to right across the f-block, which simultaneously causes a contraction and lowering of energy of the 5f orbitals and a decrease in ionic radius of the actinide elements across the series.^36,37^ It is also reasonable to suggest that the Np–O bonds formed upon complexation with the POM assembly likely contain a small amount of covalent character. Though An(IV) cations typically form electrostatic interactions with POMs due to their higher charge, the lowering of energy of the 5f orbitals for Np ions is hypothesized to increase orbital overlap with the cluster unit, resulting in stronger bonds between the actinide and cluster in **7-Np(Mo**_**5**_**)**_**2**_ compared to **4-Th(Mo**_**5**_**)**_**2**_ and **5-U(Mo**_**5**_**)**_**2**_, which is evidenced experimentally by the shortening of the M–O bond lengths in **7-Np(Mo**_**5**_**)**_**2**_ compared to **4-Th(Mo**_**5**_**)**_**2**_ and **5-U(Mo**_**5**_**)**_**2**_.^36,37^ Another consequence of this phenomena in the solid-state structure of **7-Np(Mo**_**5**_**)**_**2**_ is the distance between the central μ_5_-oxo in each cluster unit. The distance is 6.812(5) Å for **7-Np(Mo**_**5**_**)**_**2**_, sitting between the M(IV) (M = Zr, Hf) and An(IV) congeners, which exhibit distances of 6.940(2) Å and 6.843(6) Å for **4-Th(Mo**_**5**_**)**_**2**_ and **5-U(Mo**_**5**_**)**_**2**_ and 6.652(3) Å and 6.643(3) Å for **2-Zr(Mo**_**5**_**)**_**2**_ and **3-Hf(Mo**_**5**_**)**_**2**_.

**Table 1:** Pertinent Bond Distances (Å) for (TBA)_2_[Np{Mo_5_O_13_(OMe)_4_NO}_2_] (**7-Np(Mo**_**5**_**)**_**2**_)^a^

Complex	2-Zr(Mo_5_)_2_	3-Hf(Mo_5_)_2_	4-Th(Mo_5_)_2_	5-U(Mo_5_)_2_	7-Np(Mo_5_)_2_
M–O	2.201	2.191	2.410	2.358	2.349
μ_5_-O−μ_5_-O	6.652	6.643	6.940	6.843	6.812
O–O	2.751	2.751	3.105	3.012	2.987
Ionic Radius^42^	0.84	0.83	1.05	1.00	0.98

a Distances for **2-Zr(Mo**_**5**_**)**_**2**_, **3-Hf(Mo**_**5**_**)**_**2**_, **4-Th(Mo**_**5**_**)**_**2**_, and **5-U(Mo**_**5**_**)**_**2**_ are included for comparison.^31^

Structurally characterized Np–POM sandwich complexes are rare and most have been synthesized in water.^6,18^ The majority leverage Lindqvist or Keggin anions that feature tungsten as the framework metal. For example, the structurally characterized polyoxotungstoneptunates [Np(W_5_O_18_)_2_]^8–^ and [Np(SiW_11_O_39_)_2_]^12–^ feature average Np–O bond distances of 2.34 and 2.35 Å, respectively, which are either similar or identical to **7-Np(Mo**_**5**_**)**_**2**_.^6^ Moreover, Moisy and co-workers have reported the isolation of a series of An(IV) polyoxometalate clusters with the general formula [An(P_2_W_17_O_61_)_2_]^16–^ (An = Th(IV), U(IV), Np(IV), Pu(IV), Am(IV)).^18^ They observe similar bond metrics and trends (i.e., shortening of An–O contacts) due to differences in the ionic radius of the central metal cation sandwiched between the lacunary clusters.

**Figure 4:**
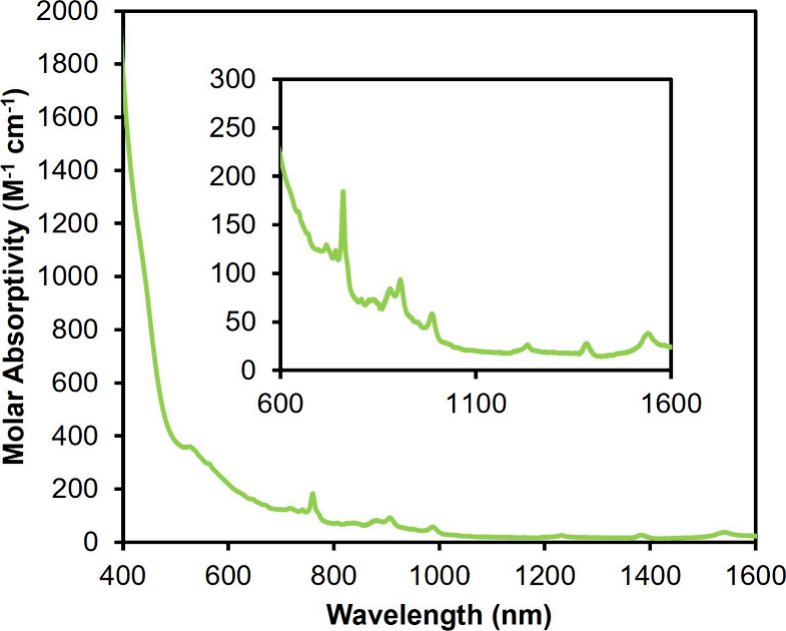
Electronic absorption spectrum of **7-Np(Mo**_**5**_**)**_**2**_ collected at room temperature in acetonitrile. The inset shows the near-infrared region of the spectrum to highlight f–f transitions of the Np(IV) center.

The pronounced difference in color between **5-U(Mo**_**5**_**)**_**2**_ (brown) and **7-Np(Mo**_**5**_**)**_**2**_ (light green) prompted our interest in characterizing **7-Np(Mo**_**5**_**)**_**2**_ via electronic absorption spectroscopy (Figure 4) to compare to the other An(IV) derivatives. The obtained UV–vis–NIR spectrum displays broad and relatively intense absorptions across the visible region, similar to that observed for the U(IV) derivative. Polyoxometalates are unique because the cluster can be treated as a singular unit that operates with a set of delocalized orbitals involving multiple metals. To this point, reports of metal-to-POM charge transfer have become increasingly common.^38−40^ In our original report, the broad absorptions in the visible region of the brown **5-U(Mo**_**5**_**)**_**2**_ cluster were tentatively assigned to uranium–molybdenum charge transfer bands given similar mechanisms of absorption have been reported for U–polyoxotungstate complexes exhibiting charge transfer character between U(IV) and W(VI).^8,17,41^ Taking into account the differences in color between the uranium and neptunium compounds, it is hypothesized that the broad and intense absorption observed from 400 to 700 nm in **7-Np(Mo**_**5**_**)**_**2**_ is a result of metal-to-ligand charge transfer (MLCT) from the neptunium metal center to the atoms within the cluster unit (Np(5f) → Mo(4d)), with additional minor contributions from ligand-to-metal charge transfer (LMCT) (O(2p)→ Np(5f)). The band at 763 nm (ε = 158 M^–1^ cm^–1^) is assigned to a Np(IV) f–f transition, and has been previously observed in reports of tetravalent neptunium coordination complexes.^42−45^ Analysis of the near-infrared region of the electronic absorbance spectrum reveals weak f–f transitions, consistent with the assignment of a 4+ oxidation state (5f^3^ electron configuration) of neptunium. Notably, **7-Np(Mo**_**5**_**)**_**2**_ has absorption bands at approximately 900 nm (ε = 92 M^–1^ cm^–1^), 990 nm (ε = 56 M^–1^ cm^–1^), 1,240 nm (ε = 24 M^–1^ cm^–1^), and 1,390 nm (ε = 25 M^–1^ cm^–1^), which are similar to other reports of tetravalent neptunium complexes and match quite well with the vis–NIR absorption spectrum of [Np(W_5_O_18_)_2_]^8–^ recorded in aqueous solution.^6,42,43,46^ The scarcity of literature detailing the electronic transitions of Np–polyoxomolybdate complexes in organic solvent highlights the importance of developing this technique to probe the electronic structure of transuranium–POM complexes.

In our previous work, cyclic voltammetry (CV) experiments showed the actinide complexes **4-Th(Mo**_**5**_**)**_**2**_ and **5-U(Mo**_**5**_**)**_**2**_ could reversibly accept up to four electrons (with the uranium derivative also showing a reversible one electron oxidation).^31^ The +4 charge of the central metal was found to be a defining factor for the observation of reversible redox events, with analogous sandwich complexes containing both Ba(II) and Bi(III) showing more complex and often irreversible redox chemistry. Furthermore, the increased size of the actinide centers was postulated to be an important factor in the stabilization of the more highly reduced states by minimizing the electrostatic repulsion between the {Mo_5_} units, with **2-Zr(Mo**_**5**_**)**_**2**_ and **3-Hf(Mo**_**5**_**)**_**2**_ showing only two reversible reduction events.

Like uranium, neptunium is redox active with access to a wide range of oxidation states between +2 and +7 (with +5 the most common in aqueous media). In acidic solutions, the Np(IV)/Np(III) couple is anodically shifted by approximately 0.7 V compared to the corresponding U(IV)/U(III) couple.^5,47^ This trend was preserved when the actinide cations were bound to polyoxometalates, with the respective An(IV)/An(III) couples of [Np(P_2_W_17_O_61_)_2_]^*n*−^ and [U(P_2_W_17_O_61_)_2_]^*n*−^ reported at −0.95 and −1.73 V.^48^ There are only a limited number of studies where Np(IV)/Np(III) redox couples are reported in nonaqueous media; similar trends of anodic shifting of the Np(IV)/Np(III) redox couple versus the corresponding U(IV)/U(III) redox couple were observed.^47,49,50^ Indeed, most recently, this was extended by La Pierre and co-workers, who reported cyclic voltammetry measurements on a series of tetravalent imidophosphorane complexes, including uranium and neptunium derivatives. Although the An(IV)/An(III) redox couple was not observed for either the Np or U derivative, reversible An(V)/An(IV) couples were observed at −0.70 V for Np and −1.57 V for U (both vs Fc^0/+^).^51^ This exemplifies how the Np(V)/Np(IV) redox couple is also anodically shifted compared to uranium, demonstrating that the trend is not specific to the An(IV)/An(III) redox couple but appears to be a generic feature of neptunium redox chemistry.

**Figure 5:**
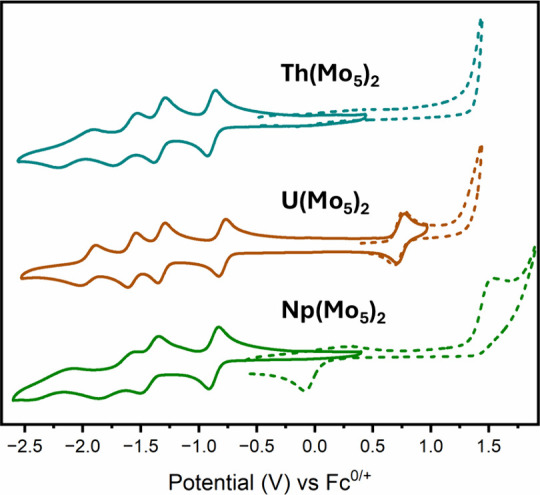
Cyclic voltammograms of **4-Th(Mo**_**5**_**)**_**2**_, **5-U(Mo**_**5**_**)**_**2**_, and **7-Np(Mo**_**5**_**)**_**2**_. Dashed traces highlight behavior at more oxidizing potentials. The data were acquired in MeCN with 0.1 M TBA(PF_6_) supporting electrolyte, 1 mM of cluster, and a scan rate of 200 mV s^–1^.

Given that the previously reported cyclic voltammogram of **5-U(Mo**_**5**_**)**_**2**_ showed the presence of a reversible U(V)/U(IV), and no U(IV)/U(III) couple, we were intrigued to determine how the redox properties of the analogous neptunium complex would compare. The cyclic voltammogram of **7-Np(Mo**_**5**_**)**_**2**_, obtained in MeCN with TBAPF_6_ as the supporting electrolyte, is shown in Figure 5. The corresponding CVs of **4-Th(Mo**_**5**_**)**_**2**_ and **5-U(Mo**_**5**_**)**_**2**_ are included for comparison (with dashed traces showing the more oxidizing regions). It is clear that **7-Np(Mo**_**5**_**)**_**2**_ possesses similar reduction properties to the other actinide containing sandwich complexes. The first two 1e^–^ reduction events of **7-Np(Mo**_**5**_**)**_**2**_ (*E*_1/2_ = −0.87, −1.43 V) appear very similar to those of **4-Th(Mo**_**5**_**)**_**2**_ (*E*_1/2_ = −0.93, −1.37 V) and **5-U(Mo**_**5**_**)**_**2**_ (*E*_1/2_ = −0.80, −1.32 V) and are assigned to addition of a single electron to each {Mo_5_} unit (i.e., Mo(VI) → Mo(V) reduction). The second two events, assigned to addition of a second electron to each of the {Mo_5_} units, are less well resolved for **7-Np(Mo**_**5**_**)**_**2**_, with larger differences between the anodic and cathodic peak potentials observed. This is indicative of a change in rate of electron transfer, likely switching from a diffusion limited process to electron transfer rate limited process. When combined with the fact that the fourth reduction event of **7-Np(Mo**_**5**_**)**_**2**_ was observed at −2.27 V (ca. 0.2–0.3 V lower than for **4-Th(Mo**_**5**_**)**_**2**_ and **5-U(Mo**_**5**_**)**_**2**_), these results indicate that the more highly reduced states of **7-Np(Mo**_**5**_**)**_**2**_ are harder to access, and may in turn be marginally less stable. This can be rationalized by the smaller size of the Np(IV) ion as compared to its early actinide counterparts; the average An–O bond length is lower for **7-Np(Mo**_**5**_**)**_**2**_ than for either **4-Th(Mo**_**5**_**)**_**2**_ or **5-U(Mo**_**5**_**)**_**2**_, leading to closer {Mo_5_} units and increased intramolecular charge repulsion. This would expectedly then place the redox characteristics of **7-Np(Mo**_**5**_**)**_**2**_ in an intermediate position between **4-Th(Mo**_**5**_**)**_**2**_**/5-U(Mo**_**5**_**)**_**2**_ and **2-Zr(Mo**_**5**_**)**_**2**_/**3-Hf(Mo**_**5**_**)**_**2**_ (which have shorter Zr–O and Hf–O bond lengths and can only be reversibly reduced by up to two electrons).

As the M(IV) ion series has been extended, a trend in the difference between the *E*_1/2_ of the first and second reduction event can be observed. The gap is smallest for **2-Zr(Mo**_**5**_**)**_**2**_/**3-Hf(Mo**_**5**_**)**_**2**_ and increases in the order **4-Th(Mo**_**5**_**)**_**2**_ < **5-U(Mo**_**5**_**)**_**2**_ < **7-Np(Mo**_**5**_**)**_**2**_. An increased gap between the first and second reduction events corresponds to a larger energy difference between the one electron and two electron reduced states. It is hypothesized that the first and second reduction events correspond to addition of a single electron to each of the {Mo_5_} units. If these {Mo_5_} units were completely electronically separated, a single two electron reduction event would be expected, as the addition of an electron to one {Mo_5_} unit would have no impact on the energy required to add an electron to the other (and therefore these energies would be expected to be the same). However, the resolution of individual redox events at different energies means that the addition of an electron to one {Mo_5_} unit affects the energy required to reduce the other. The observations here indicate some level of electronic communication between the two {Mo_5_} units and, moreover, that the gap between the energy of the first and second reduction events can be considered a measure of the extent of this communication. A larger gap, which is observed for the actinide derivatives, corresponds to stronger electronic communication between the two halves of the sandwich complex (i.e., the addition of the first electron has a stronger influence on the energy required to add the second). This may initially seem counterintuitive as the large actinide ions create a larger spatial separation between the two {Mo_5_} units. However, this observation can be rationalized by also considering that the actinide centers have access to extended valence 5f orbitals. The ability of these orbitals to overlap with the cluster based LUMO could provide a pathway for electronic communication between the two halves of the complex that is not possible for the transition metal derivatives. This is corroborated by considering that the energy of these valence 5f orbitals drops across the period, making these orbitals more accessible for U(IV) and even more so for Np(IV).^37^ This would lead to the expectation that the influence of the first reduction event on the energy of the second reduction event should increase as we move across the period, which is observed. This demonstrates that actinide substitution can be used to tune ligand based redox chemistry.

Examining the more positive region of the cyclic voltammogram of **7-Np(Mo**_**5**_**)**_**2**_ (Figure 5, green dashed line) shows the presence of an irreversible oxidation event at 1.54 V (followed by another sharp irreversible oxidation). Previously, an irreversible oxidation event has also been observed for **1-NaMo**_**5**_ (0.76 V vs Fc^0/+^ or 1.09 V vs SCE, both in MeCN), (TBA)_3_[Mo_6_O_18_NO] (0.83 V vs SCE in dimethylformamide, DMF), and (TBA)_2_[Mo_6_O_17_(OMe)NO] (1.25 V vs SCE in DMF). These oxidation events are proposed to be associated with the Mo–NO unit and are thought to shift to more positive potentials upon decreasing the overall negative charge of the system. To aid in the assignment of the oxidation events observed in the CV of **7-Np(Mo**_**5**_**)**_**2**_, the behavior at more positive potentials was also investigated for **2-Zr(Mo**_**5**_**)_2_** (Figure S16), **4-Th(Mo**_**5**_**)**_**2**_ (Figure 5, blue dashed line), and **5-U(Mo**_**5**_**)**_**2**_ (Figure 5, brown dashed line). All these compounds display a single irreversible oxidation event with an onset potential of ca. 1.35 V. Given the consistency of this event, occurring at almost identical potential for the three compounds studied, this event can confidently be assigned to the same oxidation of the Mo–NO unit previously reported, with the more anodic potentials required to drive the oxidation in **2-Zr(Mo**_**5**_**)**_**2**_, **4-Th(Mo**_**5**_**)**_**2**_, and **2-U(Mo**_**5**_**)**_**2**_ caused by the lower overall charge of the system.

This process also appears to be present in the CV of **7-Np(Mo**_**5**_**)**_**2**_, being the cause of the continued rise in current observed after the oxidation event at 1.54 V. However, the additional irreversible process is more likely to be caused by Np(IV) → Np(V) oxidation. The event occurs approximately 0.8 V higher than the previously observed, reversible, U(V)/U(IV) couple of **5-U(Mo**_**5**_**)**_**2**_. As discussed above, anodic shifting of neptunium redox couples compared to the corresponding uranium based redox couple has been commonly observed, with shifts of 0.7–0.9 V being typical, consistent with a neptunium oxidation event. Shifting of the redox couple to more positive potentials leads to a lack of stability for the oxidized species, evidenced by the irreversibility. It may be that the similarity in the potential required for Np(IV) → Np(V) oxidation to that required for oxidation of the Mo–NO unit provides a facile pathway for decomposition, with irreversible electron transfer from the Mo–NO unit to the Np(V) ion driving reformation of Np(IV) and decomposition (or rearrangement) of the sandwich complex.

To gain further insights into the chemical environments of the oxygen nuclei in our series of M(IV)/M(V) complexes and the influence of incorporation of paramagnetic ions on NMR chemical shifts, analysis by ^17^O NMR spectroscopy was pursued. ^17^O NMR spectroscopy is a powerful technique that provides detailed information about structure, bonding, and dynamic processes present in solution.^52^ Typically, ^17^O NMR chemical shifts can be correlated with the degree of M–O π-bonding (M being the framework metal), with higher π-bond order (or decreased M–O bond length) resulting in more positive chemical shifts.^52^ This leads to characteristic chemical shifts for various types of metal(VI) oxo groups, with terminal M=O groups occurring between 700 and 1000 ppm, bridging M–O–M groups at 300–600 ppm, and central oxo moieties (i.e., μ_5_-O or μ_6_-O) between 100 and −150 ppm.^53^ These values are subject to change according to redox state, protonation, changing the framework metal, or incorporation of a heterometal, all of which can have drastic impacts on the observed chemical shifts.

One major barrier to obtaining high quality ^17^O NMR spectra is the ability to efficiently ^17^O enrich the compound of interest, with spectra obtained at natural ^17^O abundance requiring a very high concentration of the compound. This can lead to high viscosity solutions and very broad resonances. Previously, some POMs have been effectively ^17^O enriched by direct treatment with ^17^O enriched water.^52^ If the M–O bonds of the POM are sufficiently labile, this leads to a dynamic equilibrium in which O atom exchange between the oxo-groups of the POM and the added water drives statistical enrichment of the cage. If the M–O bonds of the target POM are not labile with respect to O atom exchange, then alternative methods of ^17^O enrichment must be pursued.^54−56^

To probe the ability to directly enrich this family of complexes, **4-Th(Mo**_**5**_**)**_**2**_ was stirred with 10 equiv of 40% ^17^O enriched H_2_O at 50 °C for 3 h. It was hypothesized that statistical exchange of the all oxo-groups present in the structure (i.e., Mo=O, Mo–O–Mo, Mo–O–Th, and the μ_5_-O) would lead to an overall 11% ^17^O enrichment, with the only groups not enriched being the bridging -OMe groups and the terminal NO unit. Unfortunately, inspection of the resulting ^17^O NMR spectrum (Figure S7) showed only unreacted H_2_O. This indicates that **4-Th(Mo**_**5**_**)**_**2**_ undergoes no appreciable O atom exchange under these conditions, speaking to the chemical stability (with respect to rearrangement) of the [M{Mo_5_O_13_(OMe)_4_NO}_2_]^2–^ sandwich complexes in a range of solvents. This contrasts **1-NaMo**_**5**_, which has been shown to undergo facile rearrangement to (TBA)_3_[Mo_6_O_18_NO] in solvents other than MeOH.^33^

Given the inability to directly ^17^O enrich our Th(IV) complex, it was postulated that the observed instability of **1-NaMo**_**5**_ may be indicative of the lability of this framework and therefore it might serve as a better entry point for enrichment chemistry. To test this, **1-NaMo**_**5**_ was dissolved in MeOH and 5 equiv of ^17^O enriched H_2_O were added (targeting the same 11% enrichment if all oxo groups were statistically enriched). The mixture was stirred for 3 h at 50 °C before the volatiles were removed and an ^17^O NMR spectrum was obtained (Figure S8). The spectrum contained a total of six observed resonances, with the intense resonance at −16 ppm indicative of a significant amount of residual water. To minimize this, the crude material was recrystallized (see experimental section) and then extensively dried under vacuum. The spectrum was then rerecorded (Figure 3, M = Na). First, it is apparent the resonance at −16 ppm is less intense, suggesting that a significant amount of residual water was removed. The resonance observed at 18 ppm is assigned to the central μ_5_-oxo (1 O) group of the [Mo_5_O_13_(OMe)_4_NO]^3–^ cluster. An additional resonance, observed at −39 ppm, was tentatively assigned to a H_2_O bound to the sodium cation present in the cavity of [Mo_5_O_13_(OMe)_4_NO]^3–^, suggesting the coordinated MeOH can be displaced or joined by H_2_O during the enrichment. This is consistent with previous reports, where the use of hydrated materials in the synthesis of {[Mo_5_O_13_(OMe)_4_NO][Na(L)]}^2–^ led to structures where L could be both MeOH and H_2_O.^33^ The upfield shifting of this resonance compared to free H_2_O is also consistent with the increase in the oxygen coordination number upon binding to sodium.^52^

The remaining three resonances in the spectrum are all equal intensity and therefore can be assigned to the terminal Mo=O’s (4 O), bridging Mo–O–Mo’s (4 O), and formally bridging Mo–O–Na’s (4 O). The most upfield resonance, present at 417 ppm, is in the region characteristic of bridging M–O–M oxygens and therefore is assigned as such. The two remaining resonances, at 801 and 838 ppm, respectively, are in the region characteristic of terminal M=O groups. The exact assignment of these resonances can be made based on the crystallographic determined bond lengths, which relate to the π-bond order. The average terminal Mo=O bond length in **1-NaMo**_**5**_ is ca. 1.69 Å, whereas the average Mo–O–Na bond length is ca. 1.72 Å.^33^ The slightly shorter bond lengths for the Mo=O groups should result in a slightly higher chemical shift and therefore these O atoms are assigned to the resonance at 838 ppm. As a result, the resonance at 801 ppm is attributed to the Mo–O–Na groups and, when combined with the relatively short Mo–O bond length, is indicative of a significant amount of π-character in the Mo–O bonds (i.e., could be described as Mo=O···Na).

**Figure 6:**
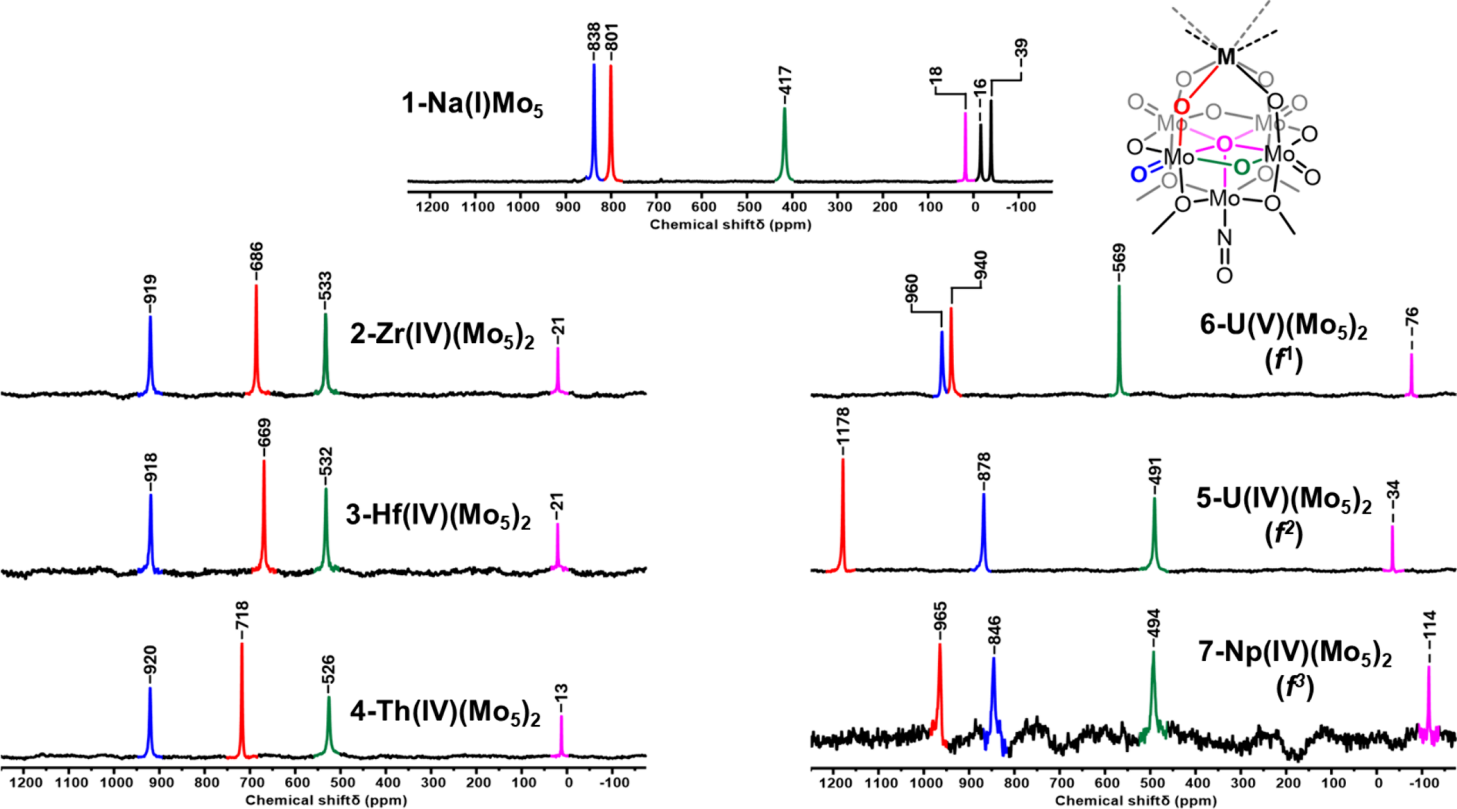
^17^O NMR spectra collected on ^17^O enriched samples of (TBA)_2_[Mo_5_O_13_(OMe)_4_NO][Na(H_2_O)] and (TBA)_*X*_[M{Mo_5_O_13_(OMe)_4_NO}_2_] sandwich complexes (when M = Zr(IV), Hf(IV), Th(IV), U(IV), or Np(IV), *X* = 2; when M = U(V), *X* = 1). Spectra obtained at 21 °C in CD_3_OD (M = Na), CDCl_3_ (M = Np), or CD_2_Cl_2_ (rest).

These results show that **1-NaMo**_**5**_ can be readily ^17^O enriched and the oxygen environments in the structure can be differentiated. This ^17^O enriched sample of **1-NaMo**_**5**_ was used in the synthesis of the M(IV)/M(V) sandwich complexes discussed both here and previously^31^ (Figure 6). The ^17^O NMR spectra of (TBA)_2_[Zr{Mo_5_O_13_(OMe)_4_NO}_2_] (**2-Zr(Mo**_**5**_**)**_**2**_) or (TBA)_2_[Hf{Mo_5_O_13_(OMe)_4_NO}_2_] (**3-Hf(Mo**_**5**_**)**_**2**_) (CD_2_Cl_2_) are very similar, mirroring the behavior observed in ^1^H NMR spectroscopy experiments. Compared to **1-NaMo**_**5**_, the resonances for the terminal Mo=O groups (blue), the bridging Mo–O–Mo groups (green), and the central μ_5_-oxo (magenta) are all shifted downfield. This can be attributed to the effective drop in charge from 2^–^ per {Mo_5_} cluster in **1-NaMo**_**5**_ to 2^–^ across two {Mo_5_} clusters in **2-Zr(Mo**_**5**_**)**_**2**_/**3-Hf(Mo**_**5**_**)**_**2**_ (effectively “deshielding” the ^17^O nuclei). Contrary to this, the resonance assigned to the Mo–O–M groups is drastically shifted upfield, occurring more than 100 ppm lower than in **1-NaMo**_**5**_. This is indicative of a decrease in the extent of π-bonding present in the Mo–O bonds, likely caused by competitive π-donation into the empty d-orbitals of the d^0^ transition metals now bound at the lacunary site of the {Mo_5_} units.^52^ This observation is consistent with the extension of the Mo–O bond lengths noted in the crystal structures of the lacunary cluster and its Zr- and Hf-congeners, going from ca. 1.72 Å in **1-NaMo**_**5**_ to ca. 1.77 Å in **2-Zr(Mo**_**5**_**)**_**2**_/**3-Hf(Mo**_**5**_**)**_**2**_.^31,33^ The observed 17 ppm difference in the position of the resonance assigned to the Mo–O–M groups in **2-Zr(Mo**_**5**_**)**_**2**_ and **3-Hf(Mo**_**5**_**)**_**2**_ indicates a minor change in the bonding between the M(IV) center and the {Mo_5_} clusters, with the lower observed chemical shift of 669 ppm signifying the Mo–O bonds of the Mo–O–Hf bridges have lower π-bond order compared to the corresponding bonds in **2-Zr(Mo**_**5**_**)**_**2**_. This could be attributed to the increased π-donation to Hf(IV) compared to Zr(IV), which is reflected in the slight shortening (ca. 0.01 Å) of the Hf–O bonds of **3-Hf(Mo**_**5**_**)**_**2**_ versus the Zr–O bonds of **2-Zr(Mo**_**5**_**)**_**2**_, likely caused by better orbital overlap between filled O 2p orbitals and empty Hf 5d oribtals which are more extended than the valence 4d orbitals of Zr.^57^

The spectrum obtained for **4-Th(Mo**_**5**_**)**_**2**_ (Figure 6) is similar to those obtained for **2-Zr(Mo**_**5**_**)**_**2**_ and **3-Hf(Mo**_**5**_**)**_**2**_. The resonances assigned to the terminal Mo=O groups (blue), bridging Mo–O–Mo groups (green), and μ_5_-oxo groups (magenta) vary only slightly from the M(IV) (M = Zr, Hf) containing derivatives, with these minor changes attributed to the installation of the larger Th(IV) cation (105 pm), which in turn leads to differences in the distances between the {Mo_5_} units of the complex. The biggest change is observed for the oxygen nuclei directly part of the thorium coordination sphere. The larger Th(IV) cation, which possesses relatively high energy (unoccupied) valence 5f orbitals, forms longer, more ionic bonds to oxygen (with π-back bonding relatively disfavored vs the Zr(IV)/Hf(IV) derivatives). Consequently, more π-electron density is available for donation to molybdenum, and therefore a downfield shift is observed for this peak, appearing now at 718 ppm for **4-Th(Mo**_**5**_**)**_**2**_ compared to 686 and 669 ppm, respectively, for **2-Zr(Mo**_**5**_**)**_**2**_ and **3-Hf(Mo**_**5**_**)**_**2**_.

More severe differences in the ^17^O NMR spectra are observed for the remaining actinide complexes (Figure 6; M = U(V) (f^1^), U(IV) (f^2^), and Np(IV) (f^3^)), which is in part caused by the presence of unpaired f-electrons in these derivatives. Though the paramagnetism of the metal ion makes complete assignment of the spectra difficult, some trends can still be observed. In particular, the resonances assigned to the terminal Mo=O groups (blue) and the bridging Mo–O–Mo (green) groups are downfield shifted in **6-U(Mo**_**5**_**)**_**2**_ (U^V^) compared to those in **5-U(Mo**_**5**_**)**_**2**_ (U^IV^) and **7-Np(Mo**_**5**_**)**_**2**_ (Np^IV^); this is most likely a result of the decrease in the overall negative charge present in the system after oxidation of U(IV) to U(V) (now only 1^–^ per two {Mo_5_} units). The remaining resonances (i.e., Mo–O–M groups and central μ_5_-O groups) appear to be less ordered. In general it can be seen that the Mo–O–M groups and central μ_5_-O groups of **5-U(Mo**_**5**_**)**_**2**_ (at 1178 and −34 ppm, respectively) appear significantly further downfield than for either **6-U(Mo**_**5**_**)**_**2**_ and **7-Np(Mo**_**5**_**)**_**2**_. This may indicate that the influence of the paramagnetic U(IV), f^2^ ion has on the surrounding ^17^O nuclei is greater than either the U(V), f^1^ or Np(IV), f^3^ ions. However, the observed paramagnetic shifting is impacted by the electronic configuration, coordination geometry, and distance of the ^17^O nuclei to the paramagnetic metal center, making rationalization of the observed shifts difficult. This is in line with the obtained ^1^H NMR spectra, where **6-U(Mo**_**5**_**)**_**2**_ and **7-Np(Mo**_**5**_**)**_**2**_ feature a single resonance at 4.79 and 4.73 ppm, respectively, assigned to the −OMe groups, which is close to the chemical shift observed for the diamagnetic complexes. Contrary to this, the same resonance is observed at 9.61 ppm for **5-U(Mo**_**5**_**)**_**2**_, significantly shifted from all other sandwich complexes studied. These observations may be further rationalized by the fact that spin–orbit heavy atom effects on light atom shielding (SO-HALA effects) are known to vary depending on the oxidation state of the heavy atom, with both the magnitude and sign of shielding/deshielding effects varying (though it is difficult to separate these effects from the effects of paramagnetism).^58^

## Conclusions

In summary, the synthesis of a family of M(IV) ion-centered sandwich complexes has been demonstrated, with an interesting extension of the series to a rare example of a neptunium(IV) derivative supported by a molybdenum based polyoxoalkoxide described. Compound **7-Np(Mo**_**5**_**)**_**2**_ behaves similarly to **5-U(Mo**_**5**_**)**_**2**_ in many ways, possessing both broad and intense absorption in the visible region of its electronic spectrum, assigned to Np(5f) → Mo(4d) MLCT, and the ability to reversibly accept up to four additional electrons. However, incorporation of the later actinide leads to subtle changes in these processes, exemplifying the ability to fine-tune the electronic structure of the system by varying the M(IV) ion present. The facile ^17^O enrichment of **1-NaMo**_**5**_ is also presented, with the dynamic nature of this structure in solution clearly allowing isotopic labeling of the oxo-groups under mild conditions. Using this ^17^O enriched material directly in the synthesis of target complexes allows isolation of actinide containing complexes which incorporate an additional spectroscopic handle. Analysis of the ^17^O NMR spectra of the series (TBA)_2_[M{Mo_5_O_13_(OMe)_4_NO}_2_] (M = Zr(IV), Hf(IV), Th(IV), U(IV), U(V), Np(IV)) produced clearly shows the ability to extract information about the influence of charge and M–O π-bond order on the chemical shifts of the oxygen nuclei present. Focusing on the U(V) (f^1^), U(IV) (f^2^), and Np(IV) (f^3^) derivatives shows a much bigger paramagnetic contribution to the observed chemical shifts for the U(IV) derivative than for either the U(V) or Np(IV) derivatives. This mimics what is observed in the ^1^H NMR data but gives a more complete picture. Overall, this further displays the utility of this system, with both a growing number of actinide derivatives in the series and an increasing number of analytical tools at our disposal to examine them. In particular, the growing number of accessible NMR spectroscopy handles available to us makes future studies focused on improving our understanding of paramagnetic shifting caused by the incorporation of heavy f-elements appealing, analogous to the detailed work already done for organic ligands.^59^ These are ideal candidates for future systematic studies exploring both An–O bonding and the influence of the actinide present on the electronic and magnetic properties of the system.

## Supporting Information Available

NMR spectra, crystal data, CV, and *E*_1/2_ values (PDF)

## References

[ref1] Icopini G. A.; Boukhalfa H.; Neu M. P. Biological Reduction of Np(V) and Np(V) Citrate by Metal-Reducing Bacteria. Environ. Sci. Technol. 2007, 41, 2764–2769. 10.1021/es0618550. 17533836

[ref2] Iveson P. B.; Rivière C.; Guillaneux D.; Nierlich M.; Thuéry P.; Ephritikhine M.; Madic C. Selective complexation of uranium(iii) over cerium(iii) by 2,6-bis(5,6-dialkyl-1,2,4-triazin-3-yl)pyridines: ^1^H NMR and X-ray crystallography studies. Chem. Commun. 2001, 1512–1513. 10.1039/b103606h.

[ref3] Liddle S. T. The Renaissance of Non-Aqueous Uranium Chemistry. Angew. Chem., Int. Ed. 2015, 54, 8604–8641. 10.1002/anie.201412168. 26079536

[ref4] Dam H. H.; Reinhoudt D. N.; Verboom W. Multicoordinate ligands for actinide/lanthanide separations. Chem. Soc. Rev. 2007, 36, 367–377. 10.1039/B603847F. 17264937

[ref5] Arnold P. L.; Dutkiewicz M. S.; Walter O. Organometallic Neptunium Chemistry. Chem. Rev. 2017, 117, 11460–11475. 10.1021/acs.chemrev.7b00192. 28853564

[ref6] Sokolova M. N.; Andreev G. B.; Yusov A. B. First transuranium mixed-ligand polyoxometalate complex. Inorg. Chem. Commun. 2011, 14, 1089–1092. 10.1016/j.inoche.2011.03.060.

[ref7] Tourne C. M.; Tourne G. F.; Brianso M.-C. Bis(undecatungstogermanato)uranate(IV) de cesium: Cs_12_[U(GeW_11_O_39_)_2_]•13–14 H_2_O. Acta Crystallogr. Sect. B: Struct. Sci. 1980, 36, 2012–2018. 10.1107/S0567740880007832.

[ref8] Bion L.; Moisy P.; Vaufrey F.; Méot-Reymond S.; Madic C. Coordination of U^4+^ in the Complex U(P_2_W_17_O_61_)_2_^16-^ in Solid State and in Aqueous Solution. Radiochim. Acta 1997, 78, 73–82. 10.1524/ract.1997.78.special-issue.73.

[ref9] Erine E. A.; Baranov A. A.; Volkov A. Y.; Chistyakov V. M.; Timofeev G. A. Thermodynamics of actinide redox reactions in potassium phosphotungstate solutions. J. Alloys Compd. 1998, 271–273, 782–785. 10.1016/S0925-8388(98)00207-2.

[ref10] Chiang M.-H.; Williams C. W.; Soderholm L.; Antonio M. R. Coordination of Actinide Ions in Wells–Dawson Heteropolyoxoanion Complexes. Eur. J. Inorg. Chem. 2003, 2003, 2663–2669. 10.1002/ejic.200300014.

[ref11] Jeannin Y. Synthèse et étude cristallographique d’un nouveau composé de coordination asymétrique de l’uranium(IV) lié à deux ligands du type polytungstate [(H_3_Sb^III^W_17_O_59_)U^IV^(HW_5_O_18_)]^11–^. Compt. Rend. Chim. 2005, 8, 999–1004. 10.1016/j.crci.2004.11.017.

[ref12] Duval S.; Sobanska S.; Roussel P.; Loiseau T. B-α-[AsW_9_O_33_]^9–^ polyoxometalates incorporating hexanuclear uranium {U_6_O_8_}-like clusters bearing the U^IV^ form or unprecedented mixed valence U^IV^/U^VI^ involving direct U^VI^-O-U^IV^ bonding. Dalton Trans. 2015, 44, 19772–19776. 10.1039/C5DT02932E. 26523545

[ref13] Dufaye M.; Duval S.; Hirsou B.; Stoclet G.; Loiseau T. Complexation of tetravalent uranium cations by the As_4_W_40_O_140_ cryptand. CrystEngComm 2018, 20, 5500–5509. 10.1039/C8CE00873F.

[ref14] Dickman M. H.; Gama G. J.; Kim K.-C.; Pope M. T. The structures of europium(III)- and uranium(IV) derivatives of [P_5_W_30_O_110_]^15^-: Evidence for “cryptohydration. J. Cluster Sci. 1996, 7, 567–583. 10.1007/BF01165802.

[ref15] Griffith W. P.; Morley-Smith N.; Nogueira H. I. S.; Shoair A. G. F.; Suriaatmaja M.; White A. J. P.; Williams D. J. Studies on polyoxo and polyperoxo-metalates: Part 7. Lanthano- and thoriopolyoxotungstates as catalytic oxidants with H_2_O_2_ and the X-ray crystal structure of Na_8_[ThW_10_O_36_]·28H_2_O. J. Organomet. Chem. 2000, 607, 146–155. 10.1016/S0022-328X(00)00308-9.

[ref16] Ostuni A.; Bachman R. E.; Pope M. T. Multiple Diastereomers of [M^n+^(α-m-P_2_W_17_O_61_)_2_]^(20–n)–^ (M = U^IV^, Th^IV^, Ce^III^; m = 1, 2). Syn- and Anti-Conformations of the Polytungstate Ligands in α_1_α_1_, α_1_α_2_, and α_2_α_2_ Complexes. J. Cluster Sci. 2003, 14, 431–446. 10.1023/B:JOCL.0000005074.03104.e1.

[ref17] Duval S.; Béghin S.; Falaise C.; Trivelli X.; Rabu P.; Loiseau T. Stabilization of Tetravalent 4f (Ce), 5d (Hf), or 5f (Th, U) Clusters by the [α-SiW_9_O_34_]^10–^ Polyoxometalate. Inorg. Chem. 2015, 54, 8271–8280. 10.1021/acs.inorgchem.5b00786. 26301948

[ref18] Sokolova M. N.; Fedosseev A. M.; Andreev G. B.; Budantseva N. A.; Yusov A. B.; Moisy P. Synthesis and Structural Examination of Complexes of Am(IV) and Other Tetravalent Actinides with Lacunary Heteropolyanion α_2_-P_2_W_17_O_61_^10–^. Inorg. Chem. 2009, 48, 9185–9190. 10.1021/ic900710c. 19725546

[ref19] Colliard I.; Lee J. R. I.; Colla C. A.; Mason H. E.; Sawvel A. M.; Zavarin M.; Nyman M.; Deblonde G. J. P. Polyoxometalates as ligands to synthesize, isolate and characterize compounds of rare isotopes on the microgram scale. Nat. Chem. 2022, 14, 1357–1366. 10.1038/s41557-022-01018-8. 36050378

[ref20] Korenev V. S.; Abramov P. A.; Gushchin A. L.; Stass D. V.; Babaev V. M.; Rizvanov I. K.; Sokolov M. N. Uranyl Incorporation into the Polyoxometalate Cavity. Synthesis and Characterization of [(UO_2_)_8_P_8_W_48_O_184_]^24–^. Russ. J. Inorg. Chem. 2019, 64, 1105–1114. 10.1134/S0036023619090146.

[ref21] Gaunt A. J.; May I.; Copping R.; Bhatt A. I.; Collison D.; Danny Fox O.; Travis Holman K.; Pope M. T. A new structural family of heteropolytungstate lacunary complexes with the uranyl, UO_2_^2+^, cation. Dalton Trans. 2003, 3009–3014. 10.1039/b302955g.

[ref22] Copping R.; Talbot-Eeckelaers C.; Collison D.; Helliwell M.; Gaunt A. J.; May I.; Reilly S. D.; Scott B. L.; McDonald R. D.; Valenzula O. A.; et al. Probing the 5f electrons in a plutonyl(vi) cluster complex. Dalton Trans. 2009, 5609–5611. 10.1039/b908648j. 20449073

[ref23] Gaunt A. J.; May I.; Helliwell M.; Richardson S. The First Structural and Spectroscopic Characterization of a Neptunyl Polyoxometalate Complex. J. Am. Chem. Soc. 2002, 124, 13350–13351. 10.1021/ja028005e. 12418864

[ref24] Kim K.-C.; Pope M. T. Cation-Directed Structure Changes in Polyoxometalate Chemistry. Equilibria between Isomers of Bis(9-tungstophosphatodioxouranate(VI)) Complexes. J. Am. Chem. Soc. 1999, 121, 8512–8517. 10.1021/ja9909125.

[ref25] Baranov A. A.; Simakin G. A.; Kosyakov V. N.; Erin E. A.; Timofeev G. A.; Kopytov V. V.; Rykov A. G. Redox potentials of pairs of Bk^4+^/Bk^3+^, Am^4+^/Am^3+^ and Ce^4+^/Ce^3+^ in K_10_P_2_W_17_O_61_ solution at different pH values. Radiokhimiya 1981, 23, 127–129.

[ref26] Saprykin A. S.; Spitsyn V. I.; Orlova M. M.; Zhuravleva O. P.; Krot N. N. Preparation and properties of compounds of uranium and transuranium elements with non-saturated heteropolytungstates. Radiokhimiya 1978, 20, 247–252.

[ref27] Saprykin A. S.; Shilov V. P.; Spitsyn V. I.; Krot N. N. Stabilization of the americium, curium and terbium tetravalent state in aqueous solutions. Dokl. Akad. Nauk SSSR 1976, 226, 4.

[ref28] Dufaye M.; Duval S.; Loiseau T. Trends and new directions in the crystal chemistry of actinide oxo-clusters incorporated in polyoxometalates. CrystEngComm 2020, 22, 3549–3562. 10.1039/D0CE00088D.

[ref29] Budantseva N. A.; Grigor’ev M. S.; Fedoseev A. M. Synthesis and spectra of Np(V) γ-Octamolybdates of the composition M_6_[(NpO_2_)_2_(Mo_8_O_28_)]·2H_2_O (M = NH_4_, K, Rb, Cs, Tl). Radiochemistry 2015, 57, 225–232. 10.1134/S1066362215030017.

[ref30] Grigorev M. S.; Charushnikova I. A.; Fedoseev A. M. Molybdate Complexes of Np(V) with Li^+^ and Na^+^ Cations in the Outer Sphere. Radiochemistry 2020, 62, 465–473. 10.1134/S1066362220040037.

[ref31] Shiels D.; Brennessel W. W.; Crawley M. R.; Matson E. M. Leveraging a reduced polyoxomolybdate-alkoxide cluster for the formation of a stable U(V) sandwich complex. Chem. Sci. 2024, 15, 11072–11083. 10.1039/D4SC02644F. 39027268 PMC11253122

[ref32] Klemperer W. G. Tetrabutylammonium Isopolyoxometalates. In Inorganic Syntheses; Ginsberg A. P. Ed.; 1990; Vol. 27, pp 74–85.

[ref33] Proust A.; Gouzerh P.; Robert F. Molybdenum oxo nitrosyl complexes. 1. Defect Lindqvist compounds of the type [Mo_5_O_13_(OR)_4_(NO)]^3-^ (R = CH_3_, C_2_H_5_). Solid-state interactions with alkali-metal cations. Inorg. Chem. 1993, 32, 5291–5298. 10.1021/ic00075a056.

[ref34] Reilly S. D.; Brown J. L.; Scott B. L.; Gaunt A. J. Synthesis and characterization of NpCl4(DME)2 and PuCl_4_(DME)_2_ neutral transuranic An(IV) starting materials. Dalton Trans. 2014, 43, 1498–1501. 10.1039/C3DT53058B. 24285347

[ref35] Shannon R. Revised effective ionic radii and systematic studies of interatomic distances in halides and chalcogenides. Acta Crystallogr. Sect. A: Found. Crystallogr. 1976, 32, 751–767. 10.1107/S0567739476001551.

[ref36] Neidig M. L.; Clark D. L.; Martin R. L. Covalency in f-element complexes. Coord. Chem. Rev. 2013, 257, 394–406. 10.1016/j.ccr.2012.04.029.

[ref37] Su J.; Batista E. R.; Boland K. S.; Bone S. E.; Bradley J. A.; Cary S. K.; Clark D. L.; Conradson S. D.; Ditter A. S.; Kaltsoyannis N.; Keith J. M.; Kerridge A.; Kozimor S. A.; Löble M. W.; Martin R. L.; Minasian S. G.; Mocko V.; La Pierre H. S.; Seidler G. T.; Shuh D. K.; Wilkerson M. P.; Wolfsberg L. E.; Yang P. Energy-Degeneracy-Driven Covalency in Actinide Bonding. J. Am. Chem. Soc. 2018, 140, 17977–17984. 10.1021/jacs.8b09436. 30540455

[ref38] Liu Y.; Wang J.; Ji K.; Meng S.; Luo Y.; Li H.; Ma P.; Niu J.; Wang J. Construction of polyoxometalate-based metal–organic frameworks through covalent bonds for enhanced visible light-driven coupling of alcohols with amines. J. Catal. 2022, 416, 149–156. 10.1016/j.jcat.2022.10.024.

[ref39] Glass E. N.; Fielden J.; Kaledin A. L.; Musaev D. G.; Lian T.; Hill C. L. Extending Metal-to-Polyoxometalate Charge Transfer Lifetimes: The Effect of Heterometal Location. Chem.—Eur. J. 2014, 20, 4297–4307. 10.1002/chem.201304119. 24604763

[ref40] Zhao C.; Huang Z.; Rodríguez-Córdoba W.; Kambara C. S.; O’Halloran K. P.; Hardcastle K. I.; Musaev D. G.; Lian T.; Hill C. L. Synthesis and Characterization of a Metal-to-Polyoxometalate Charge Transfer Molecular Chromophore. J. Am. Chem. Soc. 2011, 133, 20134–20137. 10.1021/ja209360x. 22092140

[ref41] Rusu M.; Marcu G.; Rusu D.; Roşu C.; Tomsa A. R. Uranium(IV) polyoxotungstophosphates. J. Radioanal. Nucl. Chem. 1999, 242, 467–472. 10.1007/BF02345579.

[ref42] Staun S. L.; Stevens L. M.; Smiles D. E.; Goodwin C. A. P.; Billow B. S.; Scott B. L.; Wu G.; Tondreau A. M.; Gaunt A. J.; Hayton T. W. Expanding the Nonaqueous Chemistry of Neptunium: Synthesis and Structural Characterization of [Np(NR_2_)_3_Cl], [Np(NR_2_)_3_Cl]^−^, and [Np{N(R)(SiMe_2_CH_2_)}_2_(NR_2_)]^−^ (R = SiMe_3_). Inorg. Chem. 2021, 60, 2740–2748. 10.1021/acs.inorgchem.0c03616. 33539075

[ref43] Grödler D.; Sperling J. M.; Rotermund B. M.; Scheibe B.; Beck N. B.; Mathur S.; Albrecht-Schönzart T. E. Neptunium Alkoxide Chemistry: Expanding Alkoxides to the Transuranium Elements. Inorg. Chem. 2023, 62, 2513–2517. 10.1021/acs.inorgchem.2c04338. 36705531

[ref44] Pattenaude S. A.; Anderson N. H.; Bart S. C.; Gaunt A. J.; Scott B. L. Non-aqueous neptunium and plutonium redox behaviour in THF–access to a rare Np(iii) synthetic precursor. Chem. Commun. 2018, 54, 6113–6116. 10.1039/C8CC02611D. 29736543

[ref45] Dutkiewicz M. S.; Farnaby J. H.; Apostolidis C.; Colineau E.; Walter O.; Magnani N.; Gardiner M. G.; Love J. B.; Kaltsoyannis N.; Caciuffo R.; Arnold P. L. Organometallic neptunium(III) complexes. Nat. Chem. 2016, 8, 797–802. 10.1038/nchem.2520. 27442286

[ref46] Su J.; Cheisson T.; McSkimming A.; Goodwin C. A. P.; DiMucci I. M.; Albrecht-Schönzart T.; Scott B. L.; Batista E. R.; Gaunt A. J.; Kozimor S. A.; et al. Complexation and redox chemistry of neptunium, plutonium and americium with a hydroxylaminato ligand. Chem. Sci. 2021, 12, 13343–13359. 10.1039/D1SC03905A. 34777753 PMC8528073

[ref47] Dutkiewicz M. S.; Apostolidis C.; Walter O.; Arnold P. L. Reduction chemistry of neptunium cyclopentadienide complexes: from structure to understanding. Chem. Sci. 2017, 8, 2553–2561. 10.1039/C7SC00034K. 28553487 PMC5431675

[ref48] Chiang M.-H.; Soderholm L.; Antonio M. R. Redox Chemistry of Actinide Ions in Wells–Dawson Heteropolyoxoanion Complexes. Eur. J. Inorg. Chem. 2003, 2003, 2929–2936. 10.1002/ejic.200300225.

[ref49] Sonnenberger D. C.; Gaudiello J. G. Cyclic voltammetric study of organoactinide compounds of uranium(IV) and neptunium(IV). Ligand effects on the M(IV)/M(III) couple. Inorg. Chem. 1988, 27, 2747–2748. 10.1021/ic00288a036.

[ref50] Sonnenberger D. C.; Gaudiello J. Synthesis and cyclic voltammetric study of bis(pentamethylcyclopentadienyl)neptunium dichloride. J. Less-Common Met. 1986, 126, 411–414. 10.1016/0022-5088(86)90350-4.

[ref51] Otte K. S.; Niklas J. E.; Studvick C. M.; Boggiano A. C.; Bacsa J.; Popov I. A.; La Pierre H. S. Divergent Stabilities of Tetravalent Cerium, Uranium, and Neptunium Imidophosphorane Complexes. Angew. Chem., Int. Ed. 2023, 62, e202306580 10.1002/anie.202306580. 37327070

[ref52] Filowitz M.; Ho R. K. C.; Klemperer W. G.; Shum W. Oxygen-17 nuclear magnetic resonance spectroscopy of polyoxometalates. 1. Sensitivity and resolution. Inorg. Chem. 1979, 18, 93–103. 10.1021/ic50191a021.

[ref53] Pascual-Borràs M.; López X.; Rodríguez-Fortea A.; Errington R. J.; Poblet J. M. ^17^O NMR chemical shifts in oxometalates: from the simplest monometallic species to mixed-metal polyoxometalates. Chem. Sci. 2014, 5, 2031–2042. 10.1039/c4sc00083h.

[ref54] Clegg W.; Elsegood M. R. J.; Errington R. J.; Havelock J. Alkoxide hydrolysis as a route to early transition-metal polyoxometalates: synthesis and crystal structures of heteronuclear hexametalate derivatives. J. Chem. Soc., Dalton Trans. 1996, 681–690. 10.1039/dt9960000681.

[ref55] Errington R. J.; Petkar S. S.; Middleton P. S.; McFarlane W.; Clegg W.; Coxall R. A.; Harrington R. W. Synthesis and Reactivity of the Methoxozirconium Pentatungstate (^n^Bu_4_N)6[{(μ-MeO)ZrW_5_O_18_}_2_]: Insights into Proton-Transfer Reactions, Solution Dynamics, and Assembly of {ZrW_5_O_18_}^2-^ Building Blocks. J. Am. Chem. Soc. 2007, 129, 12181–12196. 10.1021/ja0725495. 17877344

[ref56] Kandasamy B.; Wills C.; McFarlane W.; Clegg W.; Harrington R. W.; Rodríguez-Fortea A.; Poblet J. M.; Bruce P. G.; Errington R. J. An Alkoxido-Tin-Substituted Polyoxometalate [(MeO)SnW5O18]3–: The First Member of a New Family of Reactive {SnW_5_} Lindqvist-Type Anions. Chem.—Eur. J. 2012, 18, 59–62. 10.1002/chem.201103544. 22147370

[ref57] Martins C.; Aichhorn M.; Biermann S. Coulomb correlations in 4d and 5d oxides from first principles—or how spin–orbit materials choose their effective orbital degeneracies. J. Phys.: Condens. Matter 2017, 29, 263001 10.1088/1361-648X/aa648f. 28262638

[ref58] Vicha J.; Novotný J.; Komorovsky S.; Straka M.; Kaupp M.; Marek R. Relativistic Heavy-Neighbor-Atom Effects on NMR Shifts: Concepts and Trends Across the Periodic Table. Chem. Rev. 2020, 120, 7065–7103. 10.1021/acs.chemrev.9b00785. 32574047

[ref59] Parker D.; Suturina E. A.; Kuprov I.; Chilton N. F. How the ligand field in lanthanide coordination complexes determines magnetic susceptibility anisotropy, paramagnetic NMR shift, and relaxation behavior. Acc. Chem. Res. 2020, 53, 1520–1534. 10.1021/acs.accounts.0c00275. 32667187 PMC7467575

