# Supplementary material for: Venturing Past Uranium: Synthesis of a Np(IV) Polyoxomolybdate–Alkoxide Sandwich Complex
Source: Inorg Chem. 2024 Nov 20;63(48):22639–49. doi: 10.1021/acs.inorgchem.4c04428 (PMC11615951; doi:10.1021/acs.inorgchem.4c04428)
Supplement: Supplementary file 1 — ic4c04428_si_001.pdf [file ic4c04428_si_001.pdf]

Electronic Supporting Information

Venturing Past Uranium: Synthesis of a Np(IV) Polyoxomolybdate-alkoxide Sandwich Complex

Leyla R. Valerio[1±], Dominic Shiels[1±], Lauren M. Lopez[2], Andrew W. Mitchell[2], Matthias Zeller[2], Suzanne C. Bart*[2], Ellen M. Matson*[1]

[1] *Department of Chemistry, University of Rochester, Rochester NY 14627 USA*

[2] *H. C. Brown Laboratory, James Tarpo Jr. and Margaret Tarpo Department of Chemistry, Purdue University, West Lafayette, IN 47907, USA*

Corresponding Author Contact Information:

Suzanne C. Bart: sbart@purdue.edu

Ellen M. Matson: matson@chem.rochester.edu

Contents

1. ^{1}H NMR spectra S3

Figure S1. ^{1}H NMR spectrum (500 MHz) of $(TBA)_2[Zr\{Mo_5O_{13}(OMe)_4NO\}_2]$ (**2-Zr(Mo$_5$)$_2$**) in $CDCl_3$. S3

Figure S2. ^{1}H NMR spectrum (500 MHz) of $(TBA)_2[Hf\{Mo_5O_{13}(OMe)_4NO\}_2]$ (**3-Hf(Mo$_5$)$_2$**) in $CDCl_3$. S3

Figure S3. ^{1}H NMR spectrum (500 MHz) of $(TBA)_2[Th\{Mo_5O_{13}(OMe)_4NO\}_2]$ (**4-Th(Mo$_5$)$_2$**) in $CDCl_3$. S4

Figure S4. ^{1}H NMR spectrum (500 MHz) of $(TBA)[U^{V}\{Mo_5O_{13}(OMe)_4NO\}_2]$ (**6-U(Mo$_5$)$_2$**) in $CDCl_3$. Peaks marked with an asterisk correspond to the -OMe groups of **5-U(Mo$_5$)$_2$** and residual MeCN. S4

Figure S5. ^{1}H NMR spectrum (500 MHz) of $(TBA)_2[U^{IV}\{Mo_5O_{13}(OMe)_4NO\}_2]$ (**5-U(Mo$_5$)$_2$**) in $CDCl_3$. S5

Figure S6. ^{1}H NMR spectrum (400 MHz) of $(TBA)_2[Np^{IV}\{Mo_5O_{13}(OMe)_4NO\}_2]$ (**7-Np(Mo$_5$)$_2$**) in $CDCl_3$. S5

Figure S7. ^{17}O NMR spectrum (67.8 MHz) obtained after stirring $(TBA)_2[Th^{IV}\{Mo_5O_{13}(OMe)_4NO\}_2]$ (**4-Th(Mo$_5$)$_2$**) with 10 eq. of 40% ^{17}O enriched water for 3 hours at 50 °C in CD_3CN. S6

Figure S8. ^{17}O NMR spectrum (67.8 MHz) obtained after stirring $(TBA)_2[Mo_5O_{13}(OMe)_4NO][Na(MeOH)]$ (**1-NaMo$_5$**) with 5 eq. of 40% ^{17}O enriched water for 3 hours at 50 °C in MeOH. The solution was dried and the crude material was dissolved in CD_3OD for NMR spectroscopy. S6

Figure S9. ^{17}O NMR spectrum (67.8 MHz) of ^{17}O enriched $(TBA)_2[Mo_5O_{13}(OMe)_4NO[Na(MeOH)(H_2O)]$ after recrystallization and drying under vacuum. The spectrum was acquired in CD_3OD. S7

Figure S10. ^{17}O NMR spectrum (67.8 MHz) of ^{17}O enriched $(TBA)_2[Zr^{IV}\{Mo_5O_{13}(OMe)_4NO\}_2]$ (**2-Zr(Mo$_5$)$_2$**) in CD_2Cl_2. .... S7

Figure S11. ^{17}O NMR spectrum (67.8 MHz) of ^{17}O enriched $(TBA)_2[Hf^{IV}\{Mo_5O_{13}(OMe)_4NO\}_2]$ (**3-Hf(Mo$_5$)$_2$**) in CD_2Cl_2. .... S8

Figure S12. ^{17}O NMR spectrum (67.8 MHz) of ^{17}O enriched $(TBA)_2[Th^{IV}\{Mo_5O_{13}(OMe)_4NO\}_2]$ (**4-Th(Mo$_5$)$_2$**) in CD_2Cl_2. .... S8

Figure S13. ^{17}O NMR spectrum (67.8 MHz) of ^{17}O enriched $(TBA)[U^{V}\{Mo_5O_{13}(OMe)_4NO\}_2]$ (**6-U(Mo$_5$)$_2$**) (f^1) in CD_2Cl_2. .... S9

Figure S14. ^{17}O NMR spectrum (67.8 MHz) of ^{17}O enriched $(TBA)_2[U^{IV}\{Mo_5O_{13}(OMe)_4NO\}_2]$ (**5-U(Mo$_5$)$_2$**) (f^2) in CD_2Cl_2. .... S9

Figure S15. ^{17}O NMR spectrum (54.2 MHz) of ^{17}O enriched $(TBA)_2[Np^{IV}\{Mo_5O_{13}(OMe)_4NO\}_2]$ (**7-Np(Mo$_5$)$_2$**) (f^3) in $CDCl_3$. S10

2. Single crystal X-ray diffraction S11

Table S1. Crystallographic parameters for 7-Np(Mo$_5$)$_2$ S11

3. Electrochemistry S12

Figure S16. Cyclic voltammogram of **2-Zr(Mo$_5$)$_2$**. Dashed trace highlights behavior at more oxidizing potentials. The data was acquired in MeCN with 0.1 M $TBA(PF_6)$ supporting electrolyte, 1 mM of cluster, and a scan rate of 200 mV s^{-1}. S12

Table S2. $E_{1/2}$ of the reduction events present in the CVs of the series of $(TBA)_2[M^{IV}\{Mo_5O_{13}(OMe)_4NO\}_2]$ (M = Zr, Hf, Th, U, Np) discussed. The difference in the potentials of the 1st and 2nd reduction events (and 3rd vs 4th where appropriate) are also given. S12

1. ^{1}H NMR spectra

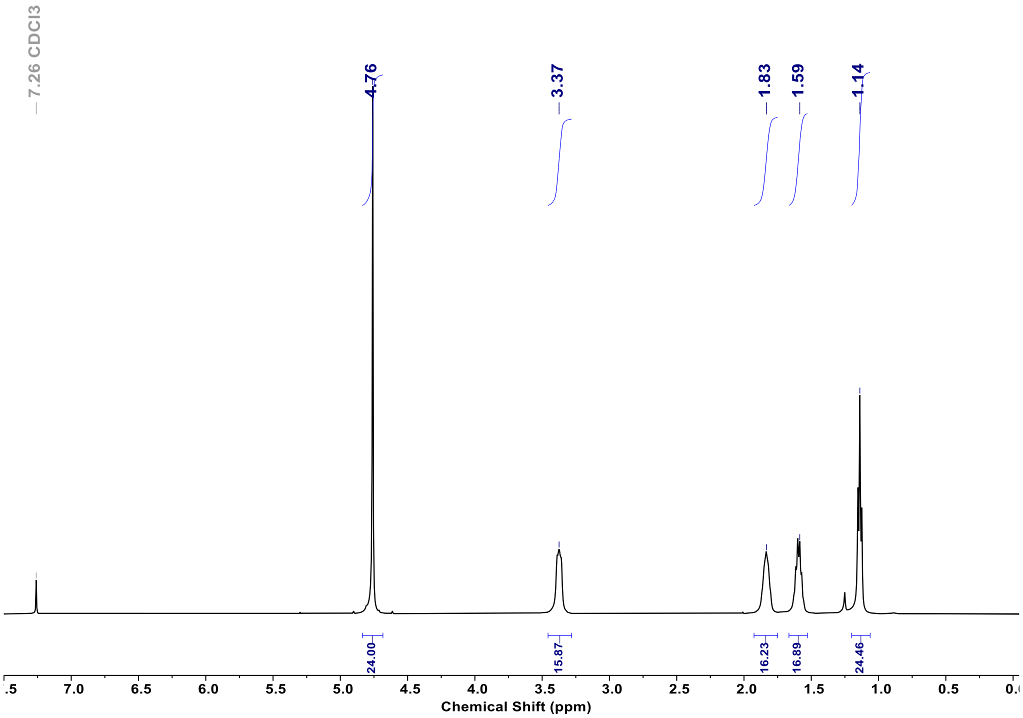

Figure S1. ^{1}H NMR spectrum (500 MHz) of $(TBA)_2[Zr\{Mo_5O_{13}(OMe)_4NO\}_2]$ (**2-Zr(Mo$_5$)$_2$**) in $CDCl_3$.

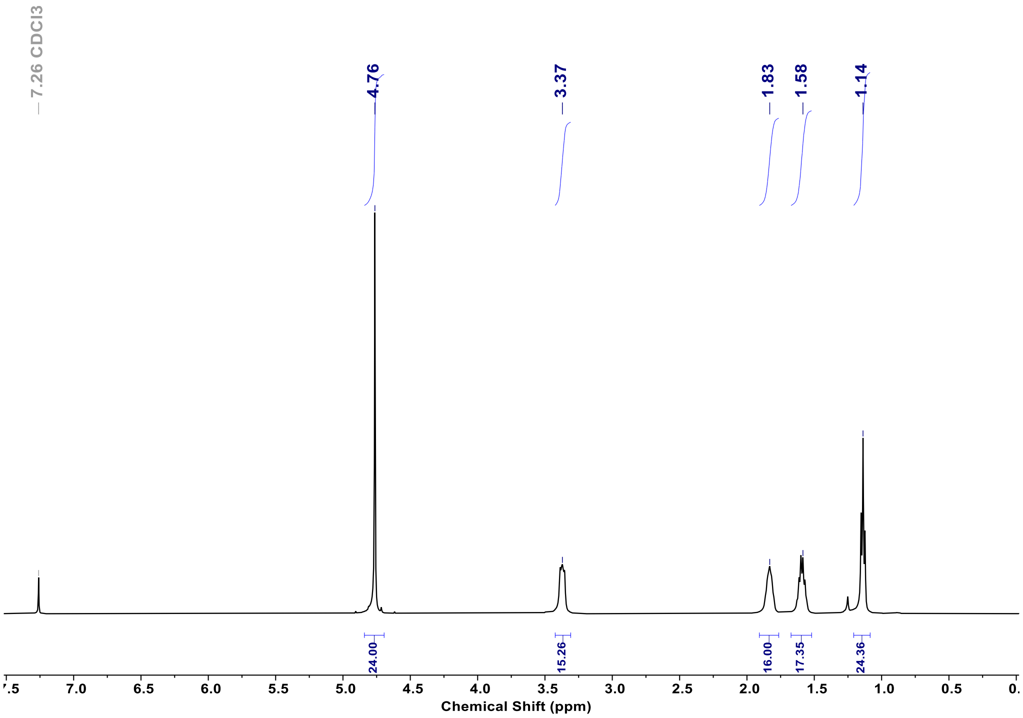

Figure S2. ^{1}H NMR spectrum (500 MHz) of $(TBA)_2[Hf\{Mo_5O_{13}(OMe)_4NO\}_2]$ (**3-Hf(Mo$_5$)$_2$**) in $CDCl_3$.

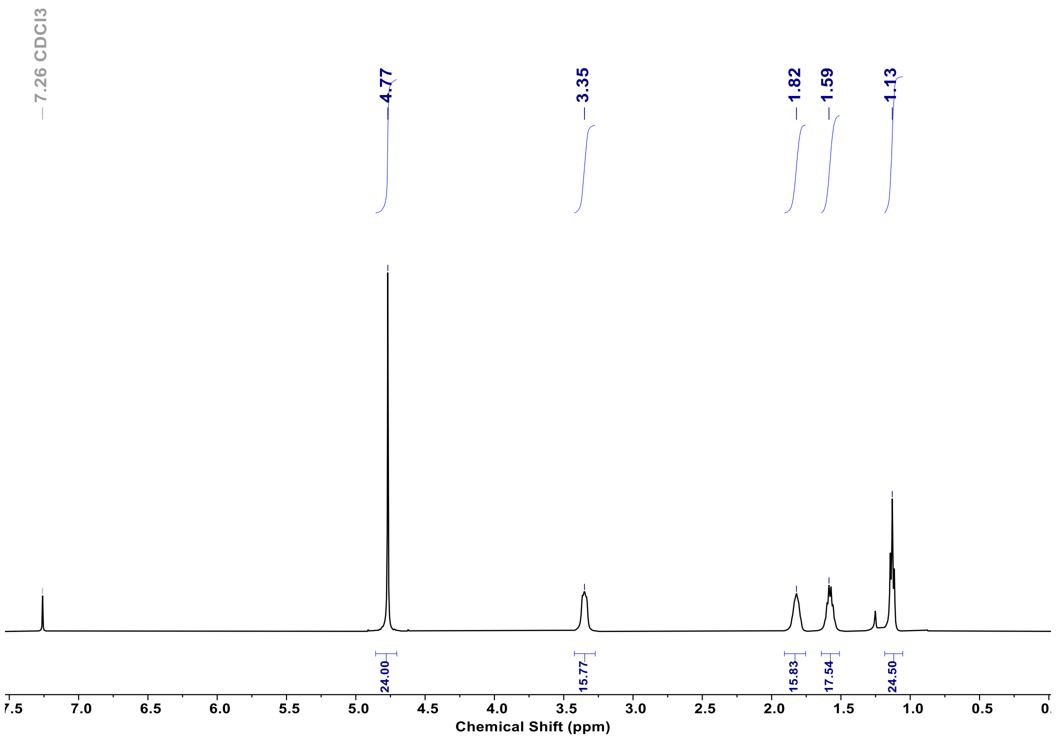

Figure S3. ^{1}H NMR spectrum (500 MHz) of $(TBA)_2[Th\{Mo_5O_{13}(OMe)_4NO\}_2]$ (**4-Th(Mo$_5$)$_2$**) in $CDCl_3$.

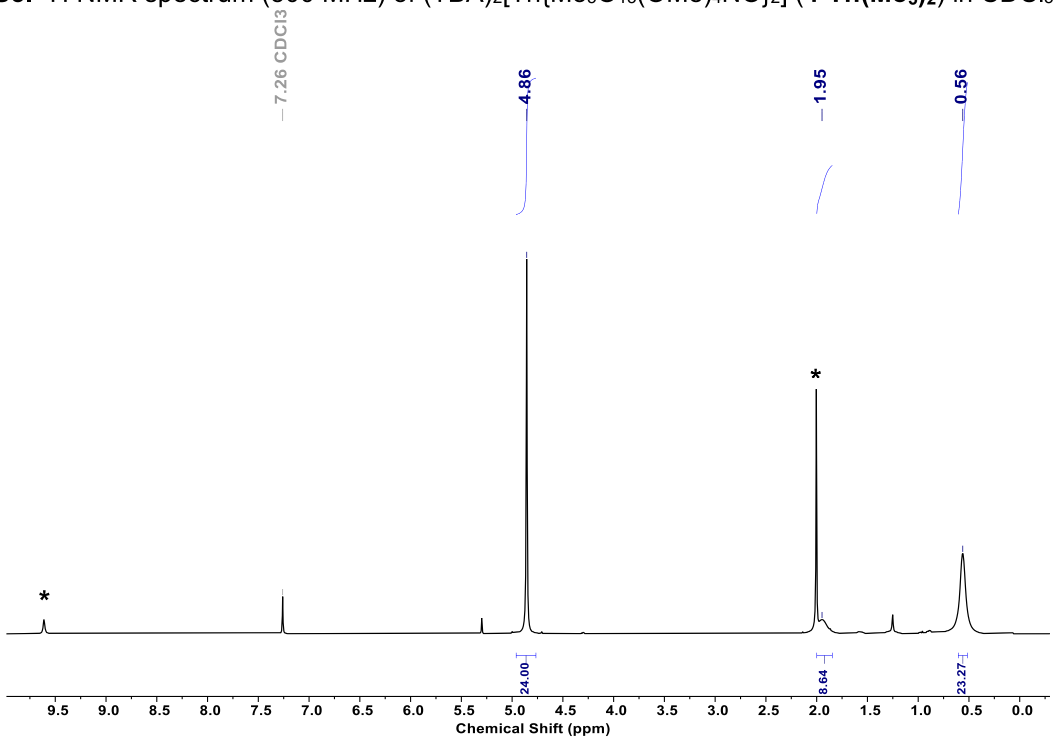

Figure S4. ^{1}H NMR spectrum (500 MHz) of $(TBA)[U^V\{Mo_5O_{13}(OMe)_4NO\}_2]$ (**6-U(Mo$_5$)$_2$**) in $CDCl_3$. Peaks marked with an asterisk correspond to the -OMe groups of **5-U(Mo$_5$)$_2$** and residual MeCN.

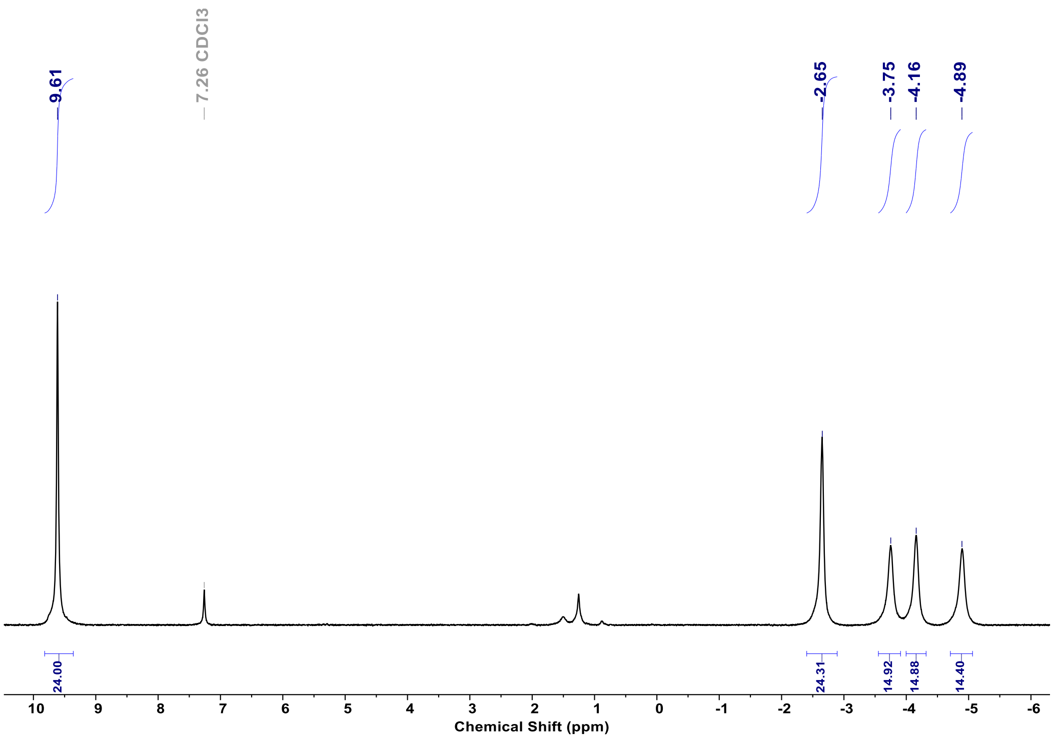

Figure S5. ^{1}H NMR spectrum (500 MHz) of $(TBA)_2[U^{IV}\{Mo_5O_{13}(OMe)_4NO\}_2]$ (**5-U(Mo$_5$)$_2$**) in $CDCl_3$.

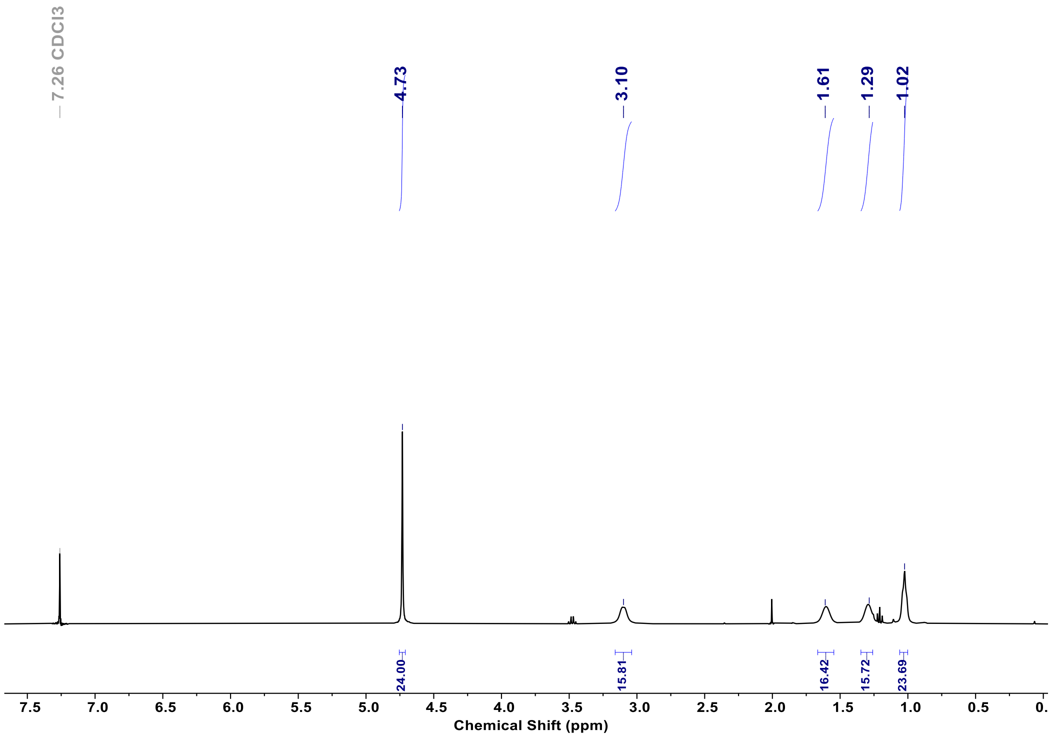

Figure S6. ^{1}H NMR spectrum (400 MHz) of $(TBA)_2[Np^{IV}\{Mo_5O_{13}(OMe)_4NO\}_2]$ (**7-Np(Mo$_5$)$_2$**) in $CDCl_3$.

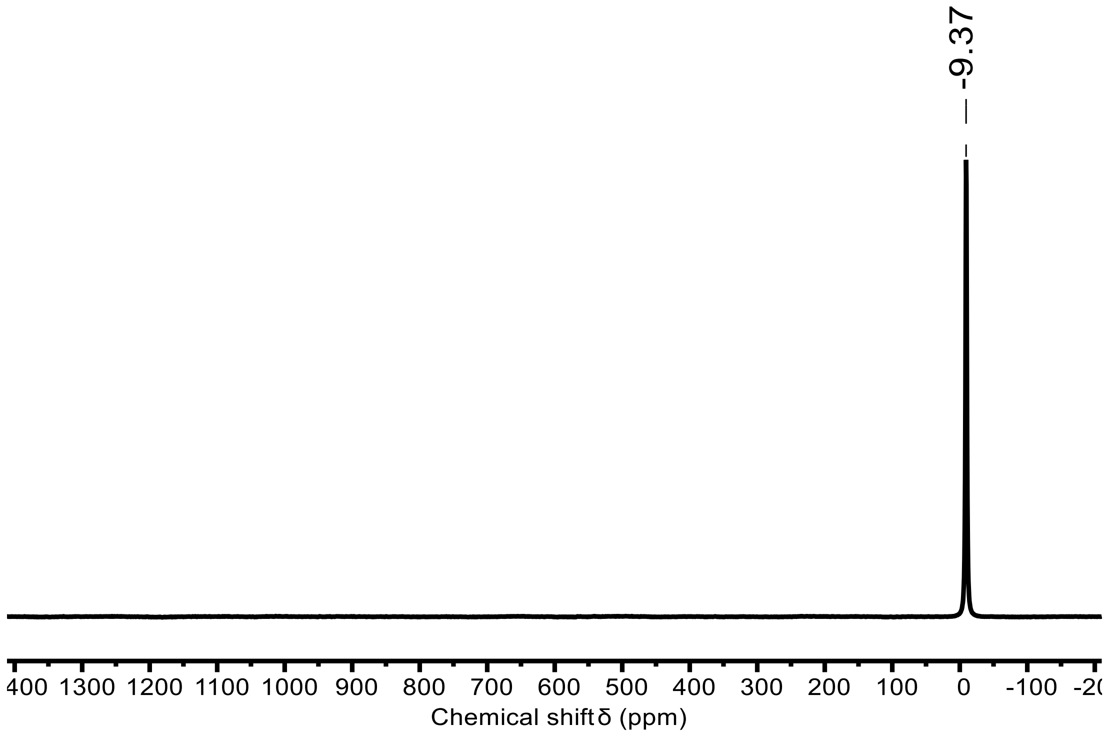

Figure S7. ^{17}O NMR spectrum (67.8 MHz) obtained after stirring $(TBA)_2[Th^{IV}\{Mo_5O_{13}(OMe)_4NO\}_2]$ (**4-Th(Mo$_5$)$_2$**) with 10 eq. of 40% ^{17}O enriched water for 3 hours at 50 °C in CD_3CN.

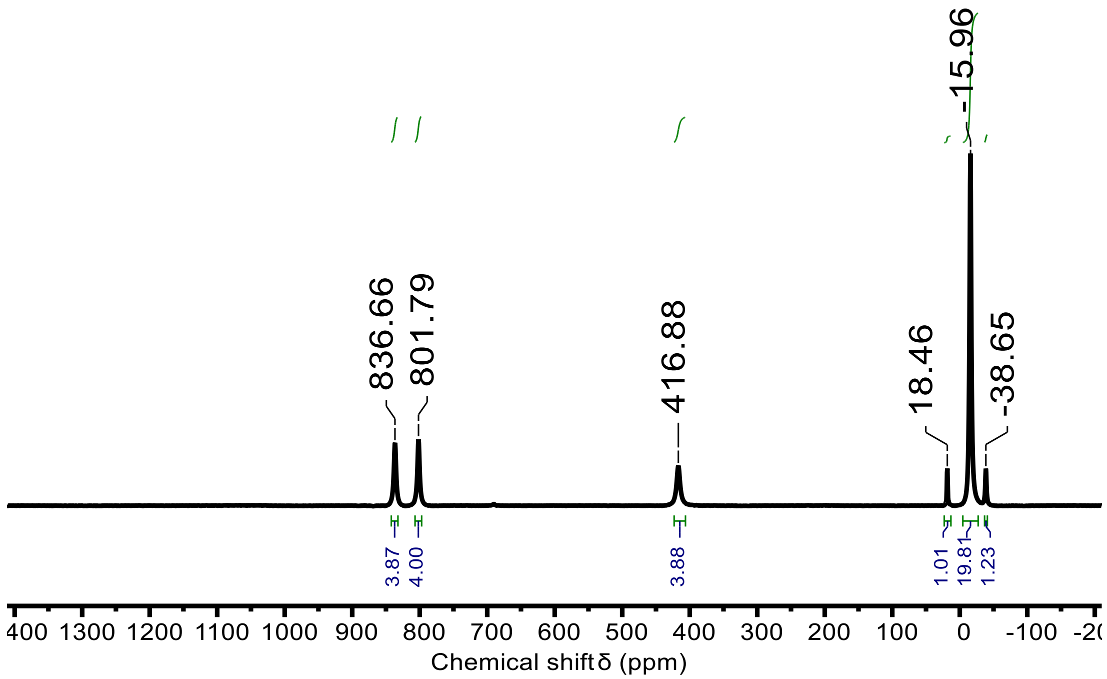

Figure S8. ^{17}O NMR spectrum (67.8 MHz) obtained after stirring $(TBA)_2[Mo_5O_{13}(OMe)_4NO][Na(MeOH)]$ (**1-NaMo$_5$**) with 5 eq. of 40% ^{17}O enriched water for 3 hours at 50 °C in MeOH. The solution was dried and the crude material was dissolved in CD_3OD for NMR spectroscopy.

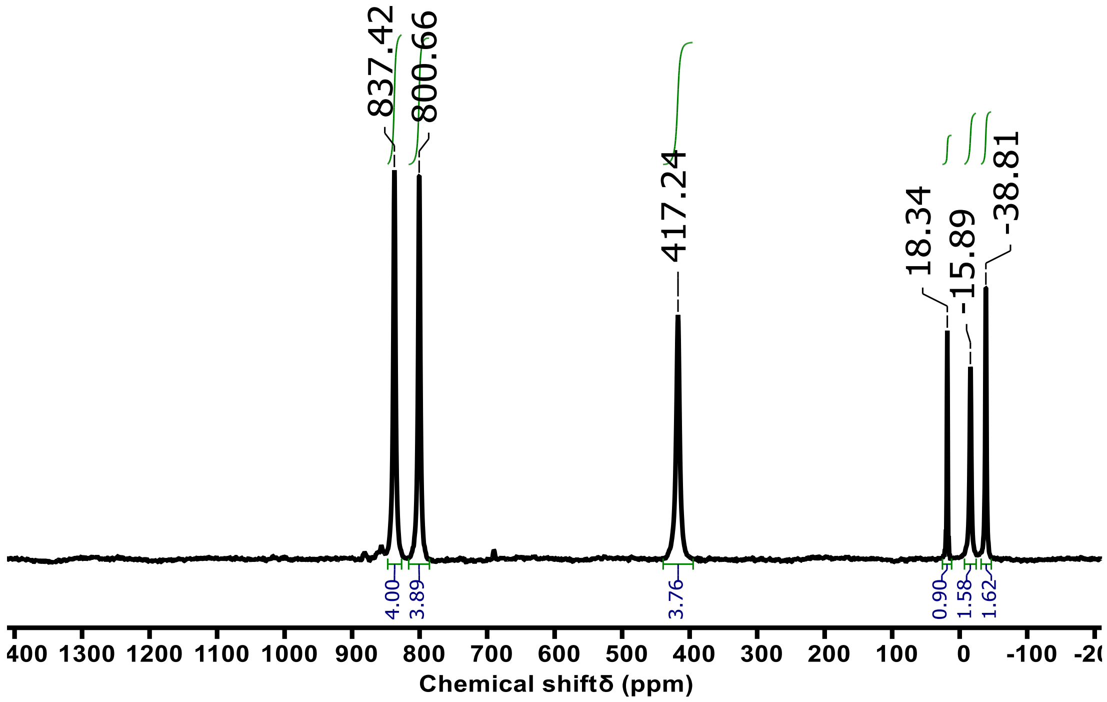

Figure S9. ^{17}O NMR spectrum (67.8 MHz) of ^{17}O enriched $(TBA)_2[Mo_5O_{13}(OMe)_4NO[Na(MeOH)(H_2O)]$ after recrystallization and drying under vacuum. The spectrum was acquired in CD_3OD.

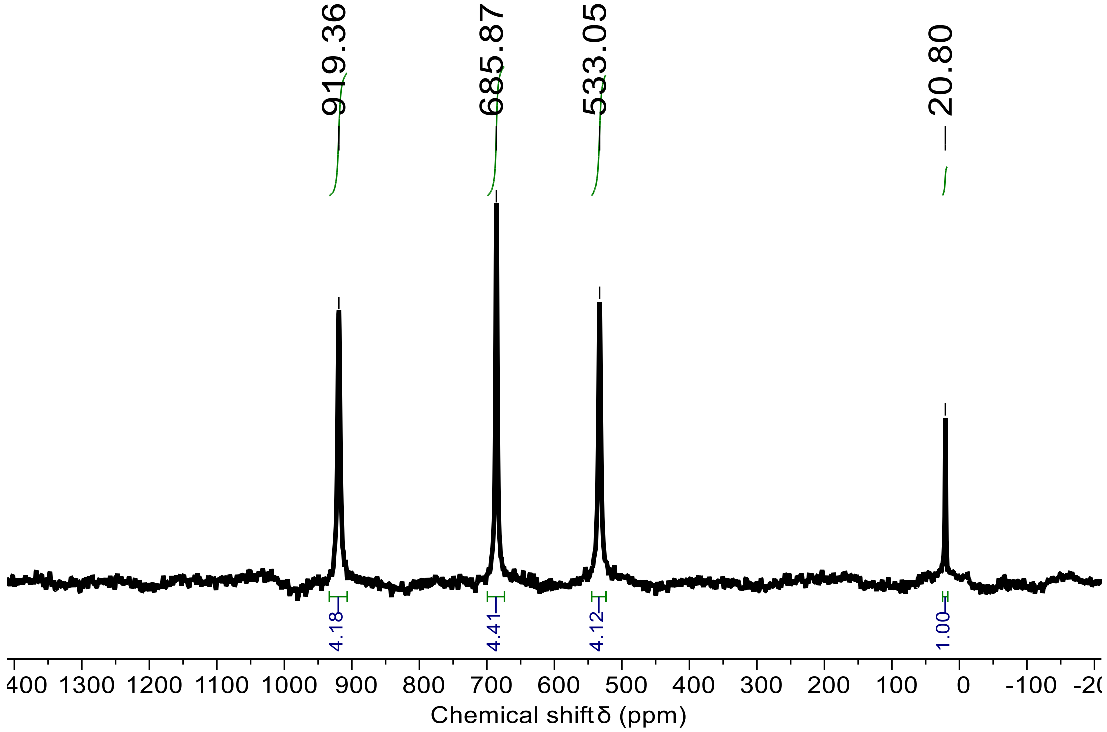

Figure S10. ^{17}O NMR spectrum (67.8 MHz) of ^{17}O enriched $(TBA)_2[Zr^{IV}\{Mo_5O_{13}(OMe)_4NO\}_2]$ (**2-Zr(Mo$_5$)$_2$**) in CD_2Cl_2.

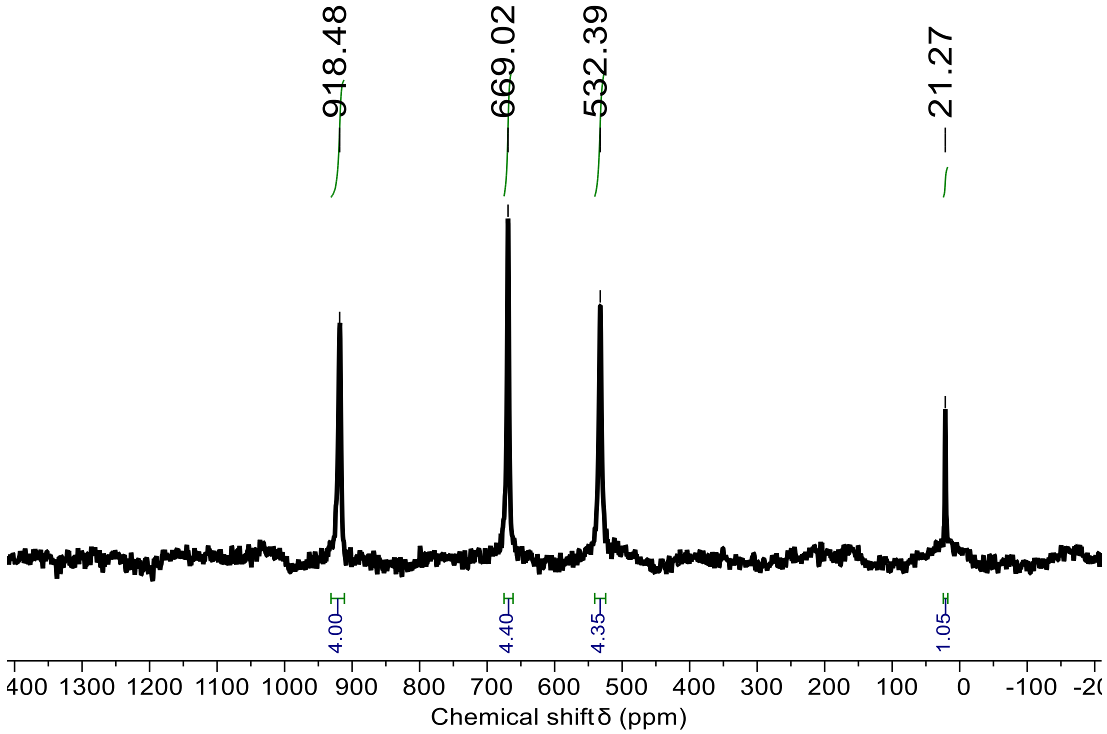

Figure S11. ^{17}O NMR spectrum (67.8 MHz) of ^{17}O enriched $(TBA)_2[Hf^{IV}\{Mo_5O_{13}(OMe)_4NO\}_2]$ (**3-Hf(Mo$_5$)$_2$**) in CD_2Cl_2.

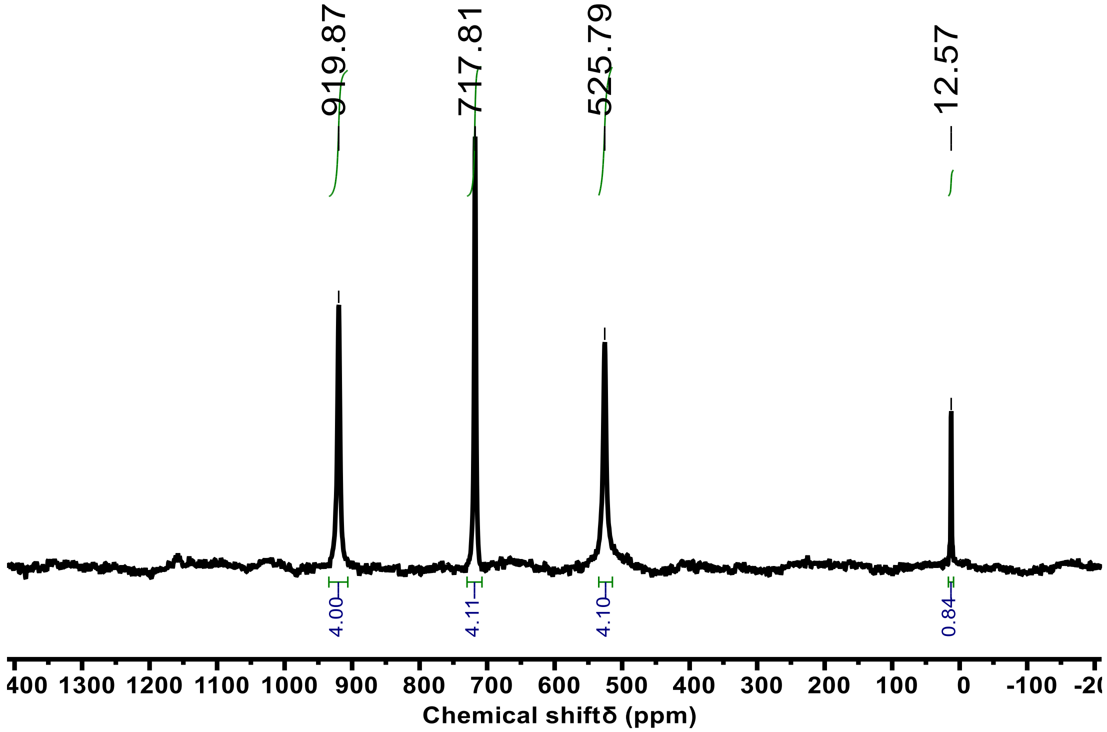

Figure S12. ^{17}O NMR spectrum (67.8 MHz) of ^{17}O enriched $(TBA)_2[Th^{IV}\{Mo_5O_{13}(OMe)_4NO\}_2]$ (**4-Th(Mo$_5$)$_2$**) in CD_2Cl_2.

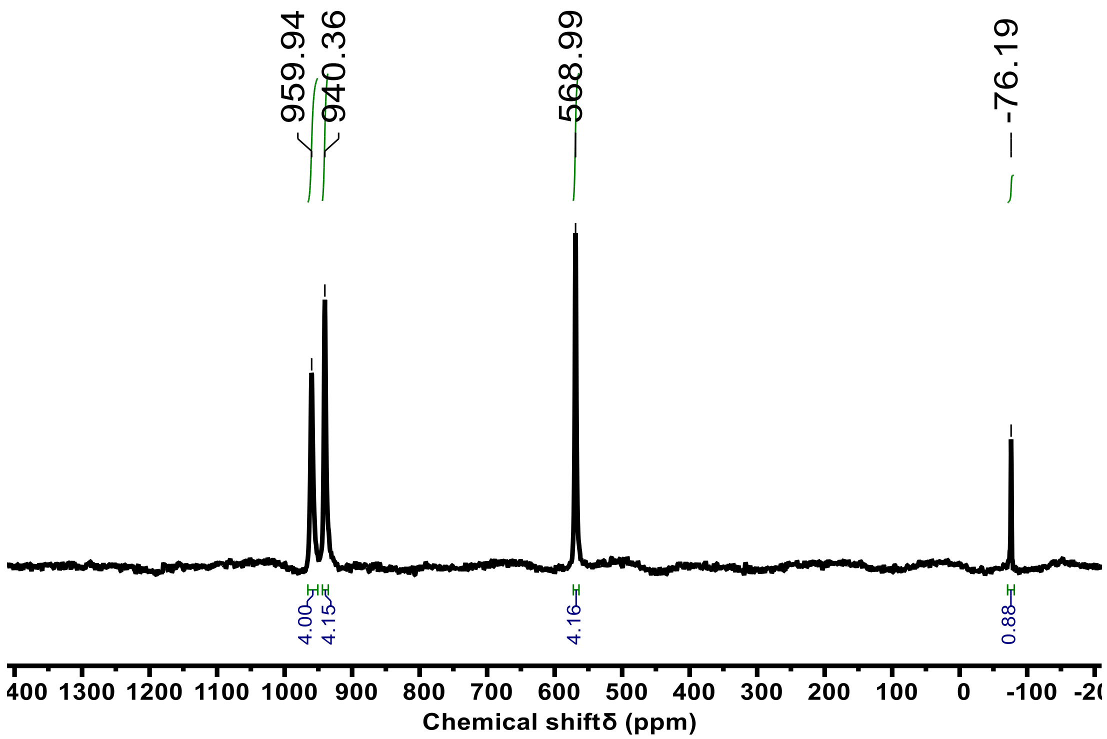

Figure S13. ^{17}O NMR spectrum (67.8 MHz) of ^{17}O enriched (TBA)[U^{V}{$Mo_5O_{13}(OMe)_4NO$}$_2$] (**6-U(Mo$_5$)$_2$**) (f^1) in CD_2Cl_2.

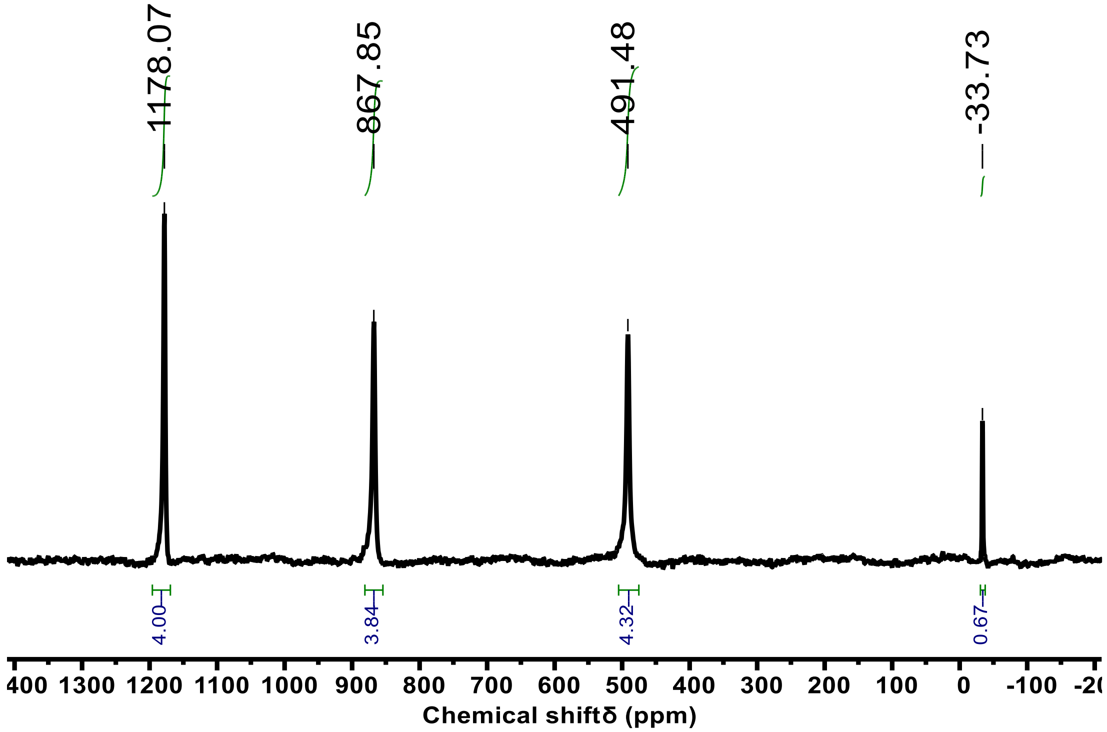

Figure S14. ^{17}O NMR spectrum (67.8 MHz) of ^{17}O enriched $(TBA)_2$[U^{IV}{$Mo_5O_{13}(OMe)_4NO$}$_2$] (**5-U(Mo$_5$)$_2$**) (f^2) in CD_2Cl_2.

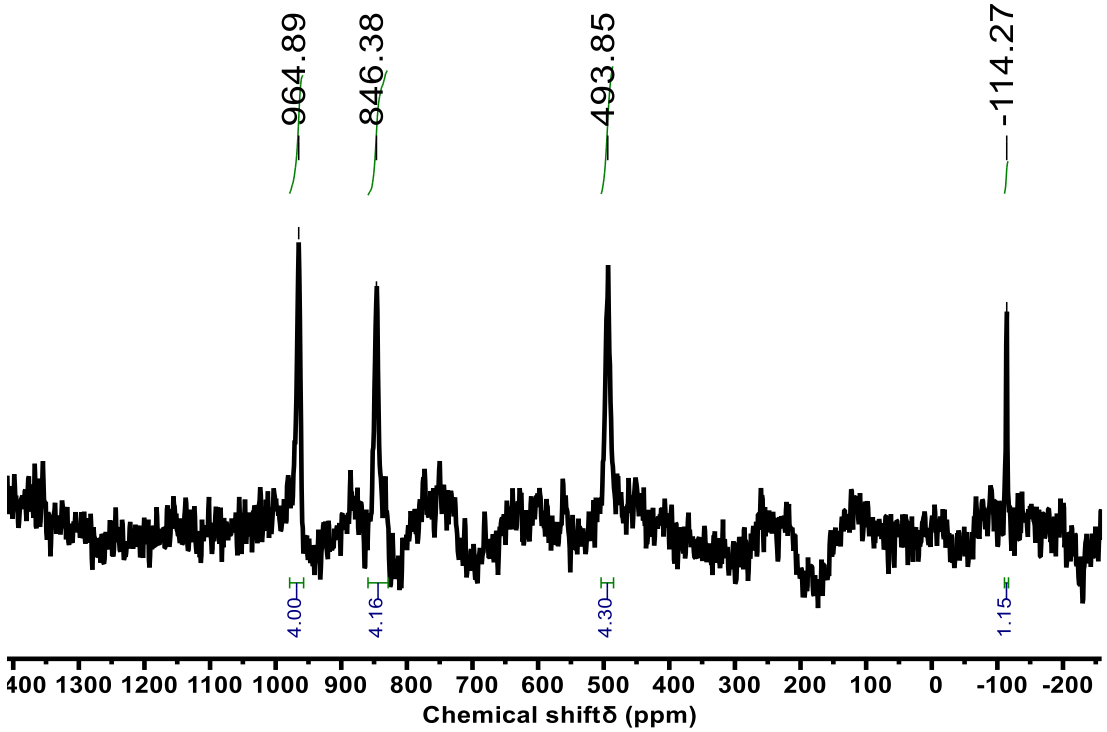

Figure S15. ^{17}O NMR spectrum (54.2 MHz) of ^{17}O enriched $(TBA)_2[Np^{IV}\{Mo_5O_{13}(OMe)_4NO\}_2]$ (**7-Np(Mo$_5$)$_2$**) (f^3) in $CDCl_3$.

2. Single crystal X-ray diffraction

Table S1. Crystallographic parameters for 7-Np(Mo_5)$_2$

**Empirical Formula**	$C_{45.12}H_{106.8}N_5O_{36.78}Mo_{10}Np$
**Formula Weight**	2504.48
**Temperature**	100(2)
**Wavelength**	MoKα (λ = 0.71073)
**Crystal System**	Monoclinic
**Space Group**	$P2_1/c$
**Unit cell Dimensions**	a = 21.7687(10)<br>b = 15.0430(6)<br>c = 24.1303(10)<br>α = 90°<br>β = 92.907(2)°<br>γ = 90°
**Volume/Å^3**	7891.7(6)
**Z**	4
**Reflections Collected**	398722
**Independent Reflections**	30148
**Goodness-of-Fit on F^2**	1.092
**Final R indices [I>2sigma(I)]**	R_1 = 0.0505, wR_2 = 0.0991

3. Electrochemistry

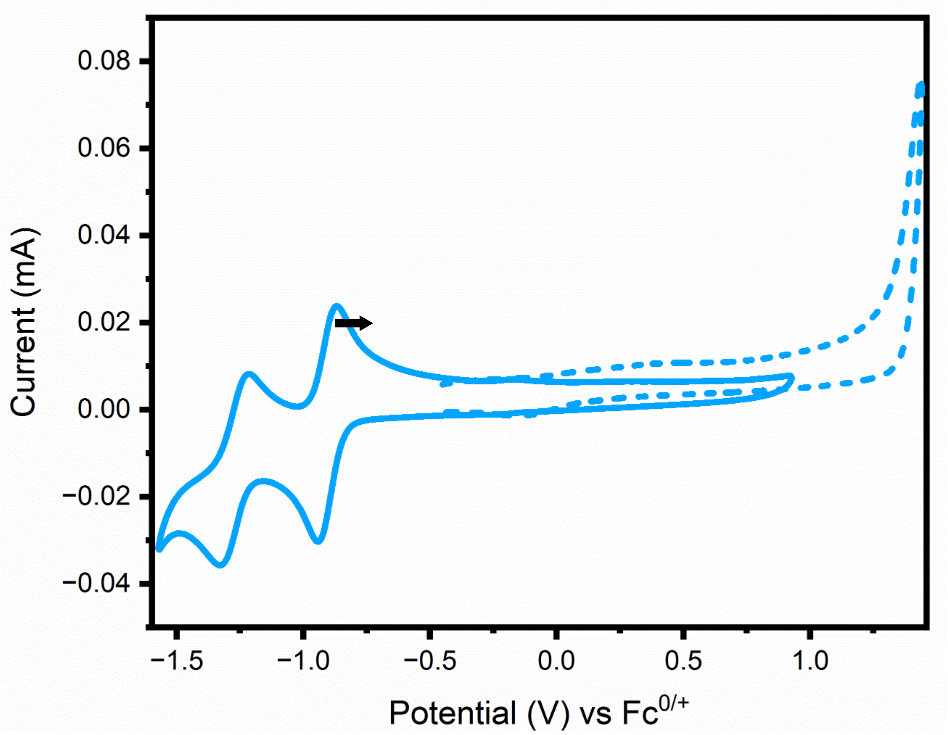

Figure S16. Cyclic voltammogram of **2-Zr(Mo$_5$)$_2$**. Dashed trace highlights behavior at more oxidizing potentials. The data was acquired in MeCN with 0.1 M TBA(PF$_6$) supporting electrolyte, 1 mM of cluster, and a scan rate of 200 mV s^{-1}.

Table S2. $E_{1/2}$ of the reduction events present in the CVs of the series of $(TBA)_2[M^{IV}\{Mo_5O_{13}(OMe)_4NO\}_2]$ (M = Zr, Hf, Th, U, Np) discussed. The difference in the potentials of the 1st and 2nd reduction events (and 3rd vs 4th where appropriate) are also given.

	$E_{1/2}$ of reduction events (V) vs $Fc^{0/+}$			
	1st	2nd	3rd	4th
**2-Zr(Mo$_5$)$_2$**	−0.905	−1.270	-	-
	$\Delta(E^{1st}/E^{2nd})$ = 0.366			
**3-Hf(Mo$_5$)$_2$**	−0.974	−1.256	-	-
	$\Delta(E^{1st}/E^{2nd})$ = 0.282			
**4-Th(Mo$_5$)$_2$**	−0.934	−1.373	−1.666	−2.076
	$\Delta(E^{1st}/E^{2nd})$ = 0.440		$\Delta(E^{3rd}/E^{4th})$ = 0.410	
**5-U(Mo$_5$)$_2$**	−0.796	−1.321	−1.575	−1.951
	$\Delta(E^{1st}/E^{2nd})$ = 0.524		$\Delta(E^{3rd}/E^{4th})$ = 0.376	
**7-Np(Mo$_5$)$_2$**	−0.874	−1.433	−1.707	−2.267
	$\Delta(E^{1st}/E^{2nd})$ = 0.559		$\Delta(E^{3rd}/E^{4th})$ = 0.560	
